# Integrated proteogenomic profiling reveals coordinated differential expression signatures during neuroinflammation

**DOI:** 10.1186/s40478-026-02365-0

**Published:** 2026-07-10

**Authors:** Elena Prepoudis, Sabina Pfister, Andreas Hofmann, Marc Altorfer, Marco Catalano, Miriam Sindelar, Pamela Ramseier, Frédérique Lafossas, Sarah Tisserand, Tobias Junt, Anna Schubart, Giuseppe Locatelli, Elizaveta Solovyeva

**Affiliations:** 1 Department of Immunology, Novartis Biomedical Research, Basel, Switzerland; 2 Department of Data & Digital, Novartis Biomedical Research, Basel, Switzerland; 3 Department of Discovery Sciences, Novartis Biomedical Research, Basel, Switzerland; 4 Department of Pharmaceutical Sciences, University of Basel, Basel, Switzerland

**Keywords:** Experimental autoimmune encephalomyelitis, Multiple sclerosis, Neuroinflammation, Spinal cord, Transcriptomics, RNA-seq, Proteomics, Proteogenomics, Mass spectrometry, Multi-omics integration

## Abstract

**Background:**

Experimental autoimmune encephalomyelitis (EAE) is a key model of autoimmune neuroinflammation, yet an integrated characterization of transcriptional and proteomic dysregulation of the CNS has been missing.

**Methods:**

In this study, we performed deep proteogenomic profiling of the spinal cord from mice induced with EAE during acute disease by combining RNA-seq (GEO, GSE330115) and LC–MS/MS (PRIDE, PXD078146).

**Results:**

We identified extensive upregulation of innate and adaptive immune response signatures alongside concordant downregulation of neuronal, synaptic, and mitochondrial pathways. Despite expected divergence as reported in previous studies discussing neuroinflammation models, log₂ fold changes and pathway enrichment scores showed high concordance between both gene product levels (R_p_ = 0.867, *p* < 2.2 × 10⁻1⁶, 95% CI [0.859, 0.874]). Loss of synaptic and metabolic integrity was predominantly observed at the protein level, whereas transcriptomics alone underestimated these structural deficits. In addition to inflammatory changes within CNS-resident cells during pathology analysis of markers typically absent in healthy CNS suggested that immune cell infiltration, in addition to pro-inflammatory phenotypic shifts of CNS-resident glial cells, accounts for the majority of non-CNS protein level changes in EAE, rather than passive plasma leakage.

**Conclusion:**

Together, this integrated dataset reveals coordinated multilayer molecular remodelling in neuroinflammation and refines mechanistic interpretation of biomarker origin in inflamed CNS tissue in mice.

**Supplementary Information:**

The online version contains supplementary material available at https://doi.org/10.1186/s40478-026-02365-0.

## Introduction

Experimental autoimmune encephalomyelitis (EAE) is a widely used model of multiple sclerosis (MS), a neuroinflammatory and neurodegenerative disease characterized by focal inflammatory lesions in the central nervous system (CNS) [1–3]. The EAE model has substantially contributed to the development of disease-modifying therapies for MS, including glatiramer acetate, natalizumab, and sphingosine-1 receptor modulators [1, 4–6].

Given its translational value, transcriptomic and proteomic dysregulation in the model have been studied intensely. Transcriptomic profiling of EAE hippocampi and retinal ganglion cells identified gene expression changes related to immune system processes, inflammatory responses, and disruption of synaptic plasticity [7–9]. Spatial transcriptomics in EAE spinal cord (SC) revealed cytokine and chemokine responses in the early disease stages, transitioning to glial cell accumulation in white matter lesions in the late stages of the disease [10]. In parallel, proteomic studies in the CNS of EAE mice have identified differentially regulated proteins impacting pathways relevant to microglial phagocytosis, blood–brain barrier (BBB) integrity, neurodegeneration, and inflammasome activation [11–13].

However, a combined large-scale multi-omics analysis integrating information from these different techniques has not been performed to date in EAE, and rarely in other inflammatory rodent models [14]. The added value of combined transcriptomic and proteomic analyses was recently exemplified in context of B cell maturation, with the discovery of ‘poised’ mRNAs in B cells that accumulate without detectable protein increases and enable rapid protein production following activation [15]. We propose that such analysis can be particularly useful in a disease model like EAE, with its multi-layered interactions between cells in the CNS parenchyma and blood-derived molecules and immune cells. Because of this interplay, an integrated analysis of tissue proteomic changes is essential to distinguish biomarkers that truly represent local pathology from those driven by systemic inflammation or BBB disruption. More broadly, these data can inform the translational relevance of the EAE model for drug discovery for MS.

This study aims to address this critical gap in our knowledge of EAE as translational research tool, by performing an integrated analysis of transcriptomic and proteomic changes in the spinal cord of EAE mice, elucidating the origin of proteins in the inflamed CNS and identifying concordant immune activation and neurodegenerative programs across molecular levels.

## Methods

### Mice

Eight-week-old female and male C57BL/6JRccHsd mice were purchased from Envigo RMS, Inc., Huntingdon, UK. Mice had ad libitum access to water and standard rodent chow. In vivo work was approved by the Veterinary Office Basel and performed according to the Swiss federal law for animal protection.

### Induction of EAE and clinical scoring

EAE was induced in two groups of age-matched mice (n = 12 for each sex) via subcutaneous injection of an emulsion containing 200 μg myelin oligodendrocyte glycoprotein (MOG_1–125_; Novartis Basel, CH; 200 μg/100 μL) emulsified in 4 mg/mL mycobacterium tuberculosis H37Ra (BD Biosciences, Franklin Lakes, USA) in Incomplete Freund's Adjuvant into the lower back. Pertussis toxin (Sigma Aldrich, St. Louis, USA) was administered intraperitoneally at 100 ng/100 μL per mouse on 0 and 2 days post-immunization (dpi). Control mice (n = 8: 4 male, 4 female) remained non-immunized. Following immunization, EAE development was scored daily with the following scoring system: 0, normal; 1, complete tail paralysis; 2, unilateral partial hind limb paralysis; 3, complete bilateral hind limb paralysis; 4, quadriplegia; 5, death.

### Selection of analysis cohort

Following EAE induction, mice were monitored daily for clinical scores and body weight. Mice were terminated at 20 dpi, i.e., 8 days after clinical onset and following the clinical peak of EAE (Additional File 1: Fig. S1). Naïve control mice (n = 8: 4 males, 4 females) were used as controls. For analysis by bulk RNA-sequencing (bulk RNA-seq) and Liquid Chromatography-Tandem Mass Spectrometry (LC–MS/MS), a subset of mice (n = 7: 4 males, 3 females) was selected. Criteria were predefined to select mice of both sexes with comparable, strong disease severity assessed by cumulative disease burden (AUC > 14.5) and serum neurofilament light chain (NfL; > 2000 pg/mL in CSF), to exclude clinical disease as a confounder. While the selection process was not blinded, it was however performed prior to biostatistical analysis.

### Sample preparation for bulk RNA-seq and LC–MS/MS

EAE mice and naïve controls were sacrificed by isoflurane overdose followed by perfusion with PBS (5 mL/min) to avoid blood contamination in the collected tissue. Spinal cords (SCs) were dissected and divided across the longitudinal midsection for bulk RNA-seq and LC–MS/MS analysis from the same animals.

SC tissue for bulk RNA-seq was collected in ice-cold RNALater (Thermo Fisher Scientific, Waltham, MA) immediately after dissection, kept at 4 °C overnight, and subsequently transferred to − 20 °C until further processing. Total RNA was extracted using the RNeasy Lipid Tissue Mini Kit (Qiagen, Hilden, DE) with DNase-I pre-treatment to remove genomic DNA contamination following the manufacturer's instructions. RNA quantity and quality were measured using the ScreenTape assay on the Agilent 4200 TapeStation system (Agilent Technologies, Santa Clara, CA). Total RNA was stored at − 80 °C until analysis.

For proteomics experiments, the other halves of SCs were homogenized in Precellys lysing kit tubes (Bertin Technologies, Montigny-le-Bretonneux, FR) containing 500 µL NHS-lysis buffer, using an Omni Bead Ruptor 24 (PreOmics, Martinsried, DE, speed = 8.00 m/s; 15 s per cycle; 4 cycles; 30 s delay between cycles). Homogenates were transferred to Protein LoBind tubes (Eppendorf, Hamburg, DE), residual beads were rinsed with 100 µL NHS-lysis buffer, and the eluent combined with the initial supernatant. For protein extraction, tissue homogenates were incubated at 95 °C for 10 min at 1000 rpm (Eppendorf ThermoMixer, Hamburg, DE). After cooling to room temperature, samples were centrifuged at 5000 × g for 2 min and protein content quantified using micro BCA assay (Thermo Fisher Scientific) and ~ 100 μg of protein were digested using TMT-iST-NHS kit (PreOmics).

### Library preparation for bulk RNA-seq

NGS libraries were prepared from 300 ng total RNA using the Illumina TruSeq Stranded mRNA Sample Preparation kit (Illumina, San Diego, CA), according to the manufacturer’s instructions (Illumina TruSeq Stranded mRNA Sample Preparation Guide, Rev. E, October 2013). The produced NGS libraries were quality controlled using the D1000 ScreenTape assay on the Agilent 4200 TapeStation system (Agilent Technologies, Santa Clara, CA). Quantification of the libraries was performed using the Qubit dsDNA HS Assay Kit (Thermo Fisher Scientific) on the Promega GloMax Explorer plate reader. Paired end 50 bp reads were generated using the Illumina NovaSeq 6000 system (Illumina), generating 44 million reads per sample on average. The sequencing data were base-called using RTA v3.4.4 software (Illumina) and processed into FASTQ file format, containing the sequence data, with the bcl2fastq2-v2.20.0.422 tool. Sequencing reads were aligned to the mm10 reference genome using the exon quantification pipeline (version 2.5) with STAR aligner (version 2.7.3a). For gene annotation, the mouse reference Gene Transfer Format (version GRCm38) from Ensembl (version NG00016.0) was used.

### TMT-based proteomics

For Tandem Mass Tag (TMT)-labelling, 100 μg of peptides from each sample were prepared using TMT-iST-NHS kit (PreOmics) and the TMT16plex reagent (Thermo Fisher Scientific), sample to channel correspondence can be found in Additional File 10: Table S11. After labelling, a small portion (4 μL) of all samples was mixed and cleaned according to the Preomics protocol. The mixed sample was dried and resuspended in 2% acetonitrile with 0.1% trifluoroacetic acid for label checking using Orbitrap Fusion Lumos mass spectrometer (Thermo Fisher Scientific) coupled with a Thermo Easy-nLC system. We used this analysis to (i) ensure labelling efficiency and (ii) to normalize the loading of channels. Labelling efficiency is estimated as the percentage of identified peptide spectrum matches (PSMs) with TMT modification to the total number of identified PSMs and was > 99%. The channel’s mixing ratio was defined as the ratio between summed intensities of all identified PSMs per channel and samples were mixed accordingly to ensure the equal amount of material per channel.

After pooling of all samples from individual mice, an Agilent1200 Series HPLC system with a Waters XBridge column (C18 3.5 μm, 150 × 2.1 mm) was used to fractionate samples into 72 fractions during a one-hour linear gradient from 0 to 50% solvent B (acetonitrile with 10 mM ammonium formate pH 11). The 72 fractions were concatenated into 24 fractions, dried, and dissolved to 0.3 μg/μl concentration (2% ACN with 0.1% formic acid) for further analysis. LC–MS analysis was carried out on an EASY-nLC 1200 system coupled to an Orbitrap Fusion Lumos Tribrid mass spectrometer (Thermo Fisher Scientific). Peptides were separated on a C18 Aurora Series UHPLC column (1.6 μm, 75 μm i.d. × 250 mm, 120 Å, Ion Opticks) using a water-acetonitrile gradient containing 0.1% formic acid for 180 min. MS1 spectra were acquired at 120 k resolution in the Orbitrap, MS2 spectra were acquired after CID activation in the ion trap and MS3 spectra were acquired after HCD activation with a synchronous precursor selection approach using five notches and 50 k resolution in the Orbitrap. LC–MS/MS raw files were analyzed with Proteome Discoverer 2.4 (Thermo Fisher Scientific). Briefly, spectra were searched with Sequest HT against the Ensemble Mus musculus GRCm38 database (68,272 entries) supplemented with custom database of common contaminates (815 proteins mostly representing keratins from several species, trypsin, and common proteins that would be found in cell culture media). The database search criteria included: 10 ppm precursor mass tolerance, 0.6 Da fragment mass tolerance, maximum three missed cleavage sites, dynamic modification of 15.995 Da for methionines, static modifications of 113.084 Da for cysteines and 304.207 Da for peptide N-termini and lysines. The Percolator algorithm was applied to the Sequest HT results. The peptide false discovery rate and the protein false discovery rate was set to 1%. TMT reporter ions of the MS3 spectra were integrated with a 20 ppm tolerance and the reporter ion intensities were used for quantification. We mitigated limited dynamic range and ratio compression by using the MS3 method and stringent interference filters to a significant degree [16].

Protein relative quantification was performed using an in-house developed R and Python script. This analysis included: (1) data filtering: exclusion of PSMs mapping to contaminants or multiple genes, exclusion of PSMs where the number of SPS mass matches were < 60%, the precursor interference was > 50% or the average reporter ion s/*n* < 10, as well as exclusion of PSMs with missing reporter ion signals; (2) global data normalization by equalizing the total reporter ion intensities across all channels, and (3) protein abundance calculation by taking mean of all PSM intensities mapping to a given gene.

### Harmonized data analysis for transcriptomics and proteomics

Transcriptomic and proteomic datasets were deposited in standard public repositories (Gene Expression Omnibus, GEO [59] / PRotein IDEntification, PRIDE [60]) and analyzed applying a harmonized workflow in R (detailed analysis parameters: Additional File 1) to ensure comparability across molecular levels. The contribution of biological covariates, including disease state, sex, and their interaction, to overall dataset variability was assessed by variation partitioning analysis (Additional File 1: Fig. S2, Tables S1-2). Differential expression was calculated separately for transcripts and proteins. For transcriptomic data, lowly expressed genes were filtered out by only keeping these with CPM > 1 in at least three biological replicates. Library sizes were normalized using the Trimmed Mean of M-values (TMM) method. Finally, the voom function was applied to compute precision weights and transform the normalized counts into log_2_ transformed values prior to linear modeling with limma. Protein intensities were normalized and log_2_ transformed prior to analysis. Linear models were fitted using limma, followed by empirical Bayes moderation, applying multiple testing using Benjamini–Hochberg correction. Gene set enrichment analysis (GSEA) was conducted using multiple pathway collections to validate reproducibility of results. While findings using WikiPathways are displayed in the main text to spotlight manually curated, biologically distinct signalling cascades [17, 18], results obtained with additional databases (Reactome, Gene Ontology Biological Processes and Molecular Functions, Hallmarks, ImmuneSigDB, MSigDB Single Cell Signatures) can be found in Additional File 3: Table S4. To facilitate biological interpretation, pathway enrichment results were consolidated to reduce redundancy.

### Statistical analysis

Statistical tests for clinical outcome assessment included paired t-tests and ordinary 1-way ANOVA (with Tukey’s or Dunnett’s post-test, respectively) using R.4.5.0. *P < 0.05; **P < 0.01; ***P < 0.001; ****P < 0.0001; ns: not significant. Correlation between transcriptomic and proteomic log2FCs was assessed using the Pearson correlation coefficient (R_p_). 95% confidence intervals (CI) were determined using Fisher’s z-transformation. To further test the significance of the correlation we used an empirical null model for permutation testing. While transcriptomic log_2_FC changes were kept constant, the corresponding proteomics log2FC values were randomly shuffled 10,000 times. A null correlation and a standard probability density function representing random chance was calculated. The empirical *p*-value was derived by calculating the proportion of the 10,000 random permutations that generated an R_p_ ≥ R_p_true.

## Results

### Molecular upregulation predominates over downregulation during EAE

**Fig. 1:**
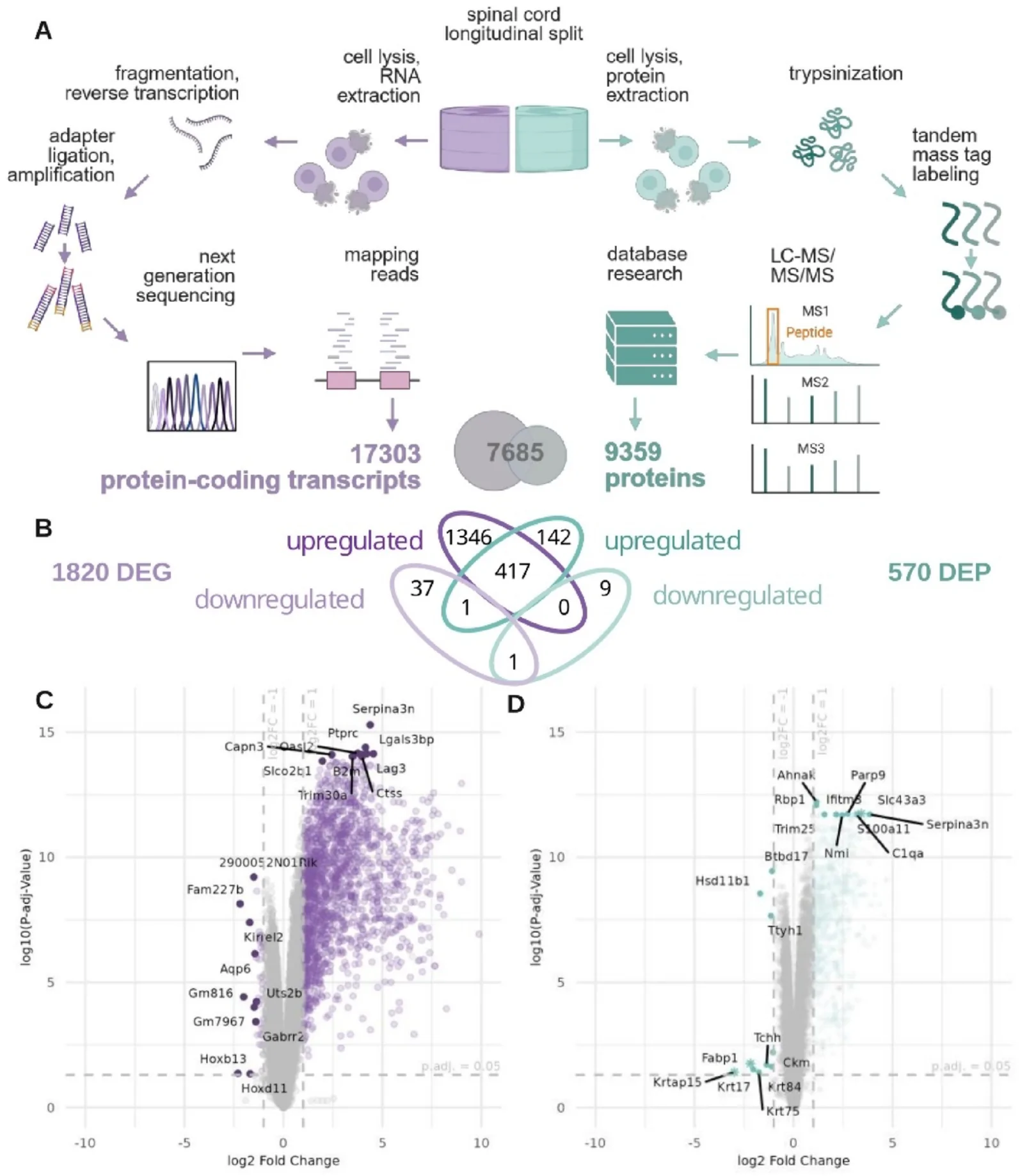
Proteogenomic profiling of EAE spinal cords reveals predominant upregulation. **A** Schematic workflow of combined transcriptomic & proteomic analysis. Spinal cord tissue from EAE & naive control mice was processed in parallel for RNA-sequencing (transcriptomics, violet) & LC–MS/MS-based profiling (proteomics, turquoise). **B** Venn diagram illustrating the count of unique & shared significantly (*p*-adj. < 0.05, log_2_FC > 1) differentially expressed transcripts/proteins (DEG/DEP). **C**–**D** Volcano plots displaying the differentially expressed transcripts (**C**) or proteins (**D**). X-axes represent the log_2_FC (EAE vs. naive), y-axes represent the significance level (log_10_ of *p*-adj.). Coloured circles denote significantly (*p*-adj. < 0.05, log_2_FC > 1) dysregulated genes, the ten candidates with highest absolute t-statistics are labelled. Proteins with significant changes but identified by only one PSM are displayed as asterisks

Spinal cords were dissected from naïve controls and EAE mice 20 dpi and cut longitudinally for paired proteomic and transcriptomic analysis from the same mice. In total, 17,303 mRNA transcripts and 9,359 proteins (Fig. 1A) were identified. Among these, 1,803 transcripts (Additional File 4: Table S5) and 570 proteins (Additional File 5: Table S6) were differentially expressed between EAE and naïve mice (|log_2_FC|> 1, *p*-adj < 0.05). This relative difference in regulation at the two molecular levels may either be caused by the limited dynamic range and ratio compression of MS–based proteomics [19, 20], or it may reflect the general tendency of proteins to exhibit smaller changes in abundance than transcripts [21]. Since both male and female mice were used, we performed variation partitioning analysis to identify potential sex differences (Additional File 1: Fig. S2). As no major contribution of sex to variance was observed and only few transcripts and proteins were differentially expressed between both sexes (0 DEG/13 DEP between naïve females and males, 5 DEG/1 DEP between EAE females and males; Additional File 1: Fig. S3, Additional File 2: Table S3), data from females and males were combined for further analysis.

EAE induction led to extensive upregulation of transcripts and proteins (1,763 or 97.7% of all 1,803 dysregulated transcripts and 560 or 98.2% of all 570 dysregulated proteins, Fig. 1B). While 74.5% (417/560) of upregulated proteins also showed significant upregulation at the transcript level, only 23.7% (417/1,763) of upregulated transcripts showed significantly increased protein levels, consistent with the lower total number of dysregulated proteins compared to transcripts. In contrast to the substantial upregulation, only 40 transcripts and 10 proteins were downregulated in EAE. Across all downregulated gene products, only one (Hsd11b1) showed concordant transcript and protein downregulation, 38 transcripts (e.g. Hoxb13, Fam227b, Gm816) and 9 proteins (e.g., Hsd11b1, Tcch, Ttyh1) were downregulated, yet were not significantly changed on the other molecular level, while one (Itih3) downregulated transcript was upregulated at the protein level.

In summary, EAE induction led to a consistent upregulation across both -omics levels, though this response was more pronounced in transcriptomics.

### Upregulation of neuroinflammation drivers is accompanied by downregulation of neurophysiology gatekeepers

To reveal the most dysregulated gene products in EAE, we ranked the degree of differential expression based on the highest absolute value of the t-statistics, a measure that considers both *p*-adj and log_2_FC (Fig. 1C, D).

The highest ranked upregulated transcripts were *B2m, Capn3, Ctss, Lag3, Lgals3bp, Oasl2, Ptprc, Serpina3n, Slco2b1,* and *Trim30a*. They are linked to antigen presentation, T cell regulation, glial reactivity, and inflammatory signalling [7, 22–29]. Conversely, the highest ranked downregulated transcripts were *2900052N01Rik, Uts2b, Aqp6, Fam227b, Gabrr2, Gm7967, Gm816, Hoxb13, Hoxd11,* and *Kirrel2*. They are relevant to B cell regulation, BBB maintenance, resting astrocytes, and neurotransmission [30–32].

The most upregulated proteins detected by more than one PSM were Ahnak, C1qa, Ifitm3, Nmi, Parp9, Rbp1, Serpina3n, Slc43a3, and Trim25. They are involved in T cell biology, antiviral defence (i.e. innate immunity), and antigen presentation [20, 25, 32–35], mirroring transcriptomic trends. The most downregulated proteins detected by more than one PSM were Btbd17, Ckm, Hsd11b1, Krt17, Krt75, Krt84, Tchh, and Ttyh1. These proteins are associated with structural and metabolic integrity, as well as neuroinflammatory processes and neuronal stem cell regulation [20, 25, 33, 36–38]. Of note, this set included three keratins which were not detected at the transcript level. While they were identified by multiple peptides and did not overlap with common contaminants (see Methods), further analyses would be required to confirm the presence of these proteins in the murine CNS.

Overall, the observed gene product upregulation involving both peripheral and CNS-specific drivers of innate and adaptive immunity is characteristic for neuroinflammation. Similarly, downregulation of neurophysiology gatekeepers is in line with the neuroinflammatory features of EAE.

### Pathway enrichment analysis confirms signature of immune activation and breakdown of CNS architecture

**Fig. 2:**
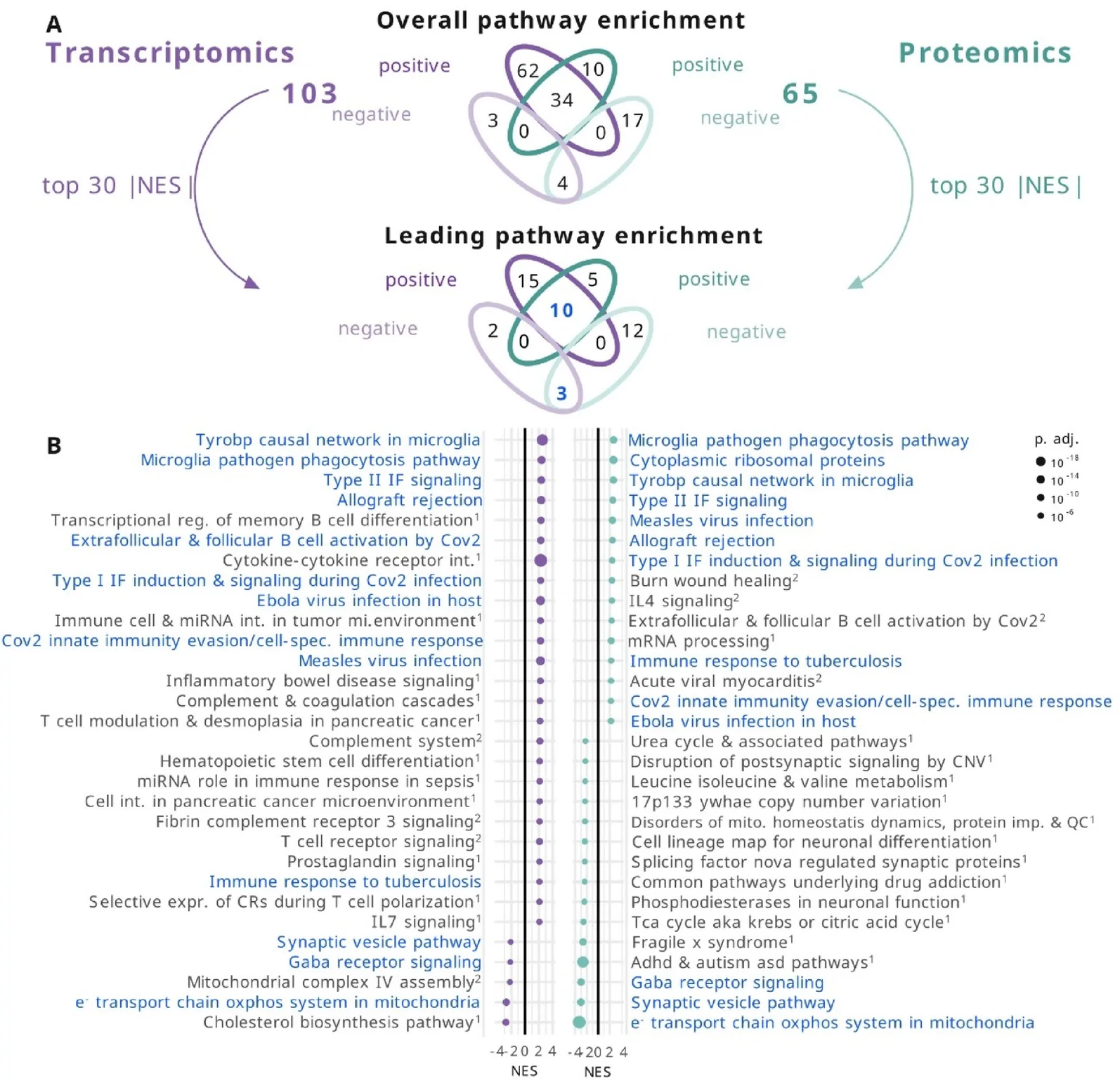
GSEA reveals concordant proteogenomic enrichment patterns. **A** Venn diagrams illustrating the count of unique and shared significantly enriched pathways (*p*-adj. < 0.05) in transcriptomics (violet) and proteomics (turquoise). **B** Top 30 enriched pathways in transcriptomics (left) or proteomics (right). X-axes represent the normalised enrichment score (NES), circle size indicates significance level. Pathways shared between the top 30 hits of both datasets are coloured to visualise convergence. Superscript numbers indicate if non-shared pathways were not enriched (^1^) at the other molecular level or enriched into the same direction (^2^)

To complement single gene evaluation with discovery of orchestrated changes at the pathway level, we performed a GSEA [39]. This revealed 103 significantly enriched pathways in transcriptomics (96 positively, 7 negatively) and 65 significantly enriched pathways in proteomics (44 positively, 21 negatively). Of note, we did not identify pathways with opposed enrichment direction, however, some were only significantly enriched at one molecular level (Fig. 2A, top).

To understand predominant enrichment signatures during EAE, we ranked pathways according to normalized enrichment scores (NES) and identified the 30 most strongly enriched pathways in both transcriptomics and proteomics (Fig. 2A, bottom). Among the top 30 leading pathways in transcriptomics, 25 were positively, while 5 were negatively enriched. Correspondingly, 15 were positively and 15 were negatively enriched in proteomics. Of the most strongly enriched pathways, 13 were shared (Fig. 2B, shown in blue), suggesting strong accordance of pathway enrichment across molecular levels.

Positive enrichment was dominated by immune signalling signatures, confirming our observations at the single gene level. The shared positively enriched pathways included mechanisms specifically leading to (I) microglia activation (*Tyrobp causal network in microglia, Microglia pathogen phagocytosis pathway*) and (II) to increase of IFNγ, IL-1β, IL-6, and TNF-α levels or associated signalling cascades like NF-κB, MAPK, and inflammasome (*Type II interferon signalling, Allograft rejection, Extrafollicular and follicular B cell activation by sarscov2, Type I interferon induction and signalling during sarscov2 infection, Ebola virus infection in host, Sarscov2 innate immunity evasion and cell-specific immune response, Measles virus infection, and Immune response to tuberculosis*), i.e. typical pathways involved in neuroinflammation. Of the remaining positively enriched pathways which only ranked among the top 30 at one molecular level (15 in transcriptomics, 5 in proteomics), 7 were significantly enriched into the same direction, but did not reach the top 30 level in both levels, whereas 13 were only significantly enriched in one molecular level. In-line with the enrichment pattern of the shared pathways, they clustered into three major functional categories: (I) innate immune activation pathways, exemplified by enrichment of pathways associated with viral defence, TLR signalling and complement system; (II) adaptive immune activation, represented by various pathways of lymphocyte differentiation and activation, cytokines and chemokines and associated receptors; (III) pathways relevant to humoral immunity and tissue remodelling.

Negative enrichment was also in-line with our findings at the single gene level and included signatures of neurodegenerative processes accompanied by stress response signalling and collapse of cellular energy supply: the shared negatively enriched pathways were relevant for synaptic signalling (*Synaptic vesicle pathway, GABA receptor signalling*) and energy metabolism (*Electron transport chain oxphos system in mitochondria*). Of the remaining negatively enriched pathways which only ranked among the top 30 at one molecular level (12 in transcriptomics, 2 in proteomics), only *Mitochondrial complex IV assembly* was also negatively enriched at the other molecular level, while 13 were not significantly enriched at the other molecular level. However, we observed functional overlap for bioenergetic pathways (e.g. *Cholesterol biosynthesis pathway, urea cycle and associated pathways*). Additional pathways exemplifying synaptic dysfunction were only present in the proteomics dataset. This highlights the neurodegenerative characteristics of EAE: synaptic dysfunction, evidenced by the downregulation of synaptic vesicle pathways, phosphodiesterase networks, and loss of neuronal maintenance signatures, as pathways associated with neuronal splicing and cytoskeletal integrity, e.g., *17p133 (YWHAE) copy number variation, Fragile X syndrome,* or *ADHD and Autism (ASD) Pathways,* were depleted.

In summary, GSEA consistently revealed a notable number of shared enriched pathways at transcript and protein level. We observed a comparable enrichment pattern for pathways involved in innate/adaptive immunity (positively enriched) and energy metabolism (negatively enriched). In addition, negative enrichment of synaptic signalling was only significant at the protein level. This may reflect insufficient metabolic resources to restore the disrupted CNS architecture during the sustained neuroinflammatory response, which imposes high metabolic demand.

### Concordant pathway enrichment during neuroinflammation

**Fig. 3:**
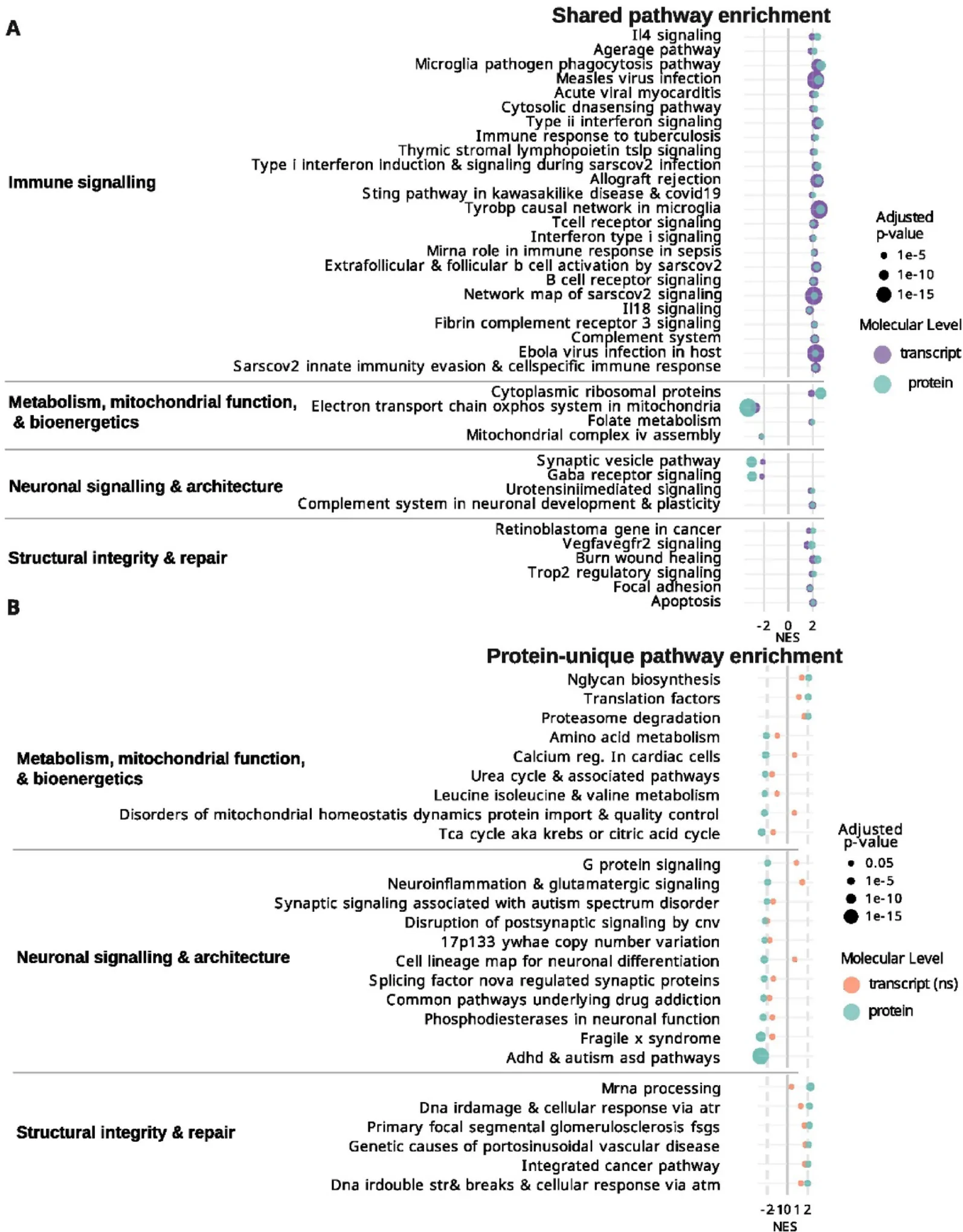
Comparative GSEA underlines concordant orchestration of the immune response alongside discordant synaptic & mitochondrial maintenance. **A** Pathways depicting strongest differences between transcriptomic (violet) & proteomic (turquoise) normalised enrichment scores (NES), sorted highest to smallest. Circle colour denotes the data type (transcript vs protein), & circle size denotes statistical significance. **B** Pathways enriched uniquely at the protein level, sorted according to highest NES. The horizontal line separates pathways ranked among top 30 from the remaining ones

To extend the comparison of changes between the two molecular levels, we next compared the degree of difference between the NES (Fig. 3A). We observed a high degree of pathway coordination with identical enrichment direction and comparable NES between the transcriptomic and proteomic datasets. While immune signalling and ribosomal regulation pathways were strongly upregulated, downregulated pathways were involved in neuronal signalling processes and mitochondrial energy supply, in-line with our evaluation of top 30 enriched pathways.

We observed a trend to higher |NES| at the protein level for pathways involved in synaptic signalling and mitochondrial biology compared to transcriptomics. In contrast to this observation, comparably lower |NES| at the protein level would be expected since proteomics analysis has lower depth compared to transcriptomics and GSEA is based on a running-sum statistic that scales with the number of detected features per pathway. Consequently, higher |NES| in proteomics would strongly suggest an increased enrichment at the protein level rather than technical artefacts. However, turnover kinetics may differ during CNS inflammation which may lead to discordances between local transcript and protein enrichment.

To evaluate which specific biological processes were predominantly enriched in proteomics, we identified pathways showing enrichment at the protein, but not at the transcript level (Fig. 3B). This pattern may reflect characteristic inflammation-driven changes: protein depletion could indicate accelerated degradation, whereas protein increases may reflect systemic influx across an impaired BBB. To ensure the technical stability of identified protein specific changes, we repeated the analysis using only proteins identified by multiple PSMs (Additional File 1: Fig. S6–S8). Overall, the results suggest the same signature pattern in both analyses.

The majority of protein-specific changes showed negative enrichment (18 negatively versus 7 positively enriched pathways). We identified three functional clusters: (I) Pathways linked to synaptic signalling and architecture, and neurological disorders with a strong link to synaptic dysfunction; (II) pathways linked to mitochondrial function and general as well as neuron-specific bioenergetics; (III) pathways linked to cytoskeletal and structural scaffolds. This pattern suggests accelerated synaptic destruction and structural damage in a glycolytic or metabolically stressed environment, matching the extensive tissue damage occurring during neuroinflammation.

Protein-specific increases were mainly relevant for cell cycle and translation, suggesting increased recruitment of tissue repair mechanisms. Furthermore, we found positive enrichment of the pathway *Primary Focal Segmental Glomerulosclerosis FSGS,* in which leading-edge proteins (Additional File 6: Table S7) are relevant for cell adhesion and cytoskeletal remodelling. Interestingly, this was accompanied by positive enrichment of *Dengue2 Interactions with Complement and Coagulation Cascades*.

Together, the comparison of pathway enrichment revealed an overall coordinated pattern between changes on transcript and protein levels. However, pathways linked to synaptic signalling exhibited greater (negative) enrichment at the protein level. Given the typically lower proteomic coverage, this finding suggests a biologically relevant impact on these pathways at the protein level.

### Proteogenomic integration reveals strong correlation of log_2_FCs

A robust understanding of local versus systemic changes of molecular biomarkers is central for the use of disease models for drug discovery. We were thus interested in discriminating proteomic changes caused by local protein dysregulation in the CNS from changes caused by protein in- or efflux across the disrupted BBB [40]. To capture both transcript-silent protein alterations and amplified protein-level dysregulation, we performed direct comparison of log_2_FCs.

**Fig. 4:**
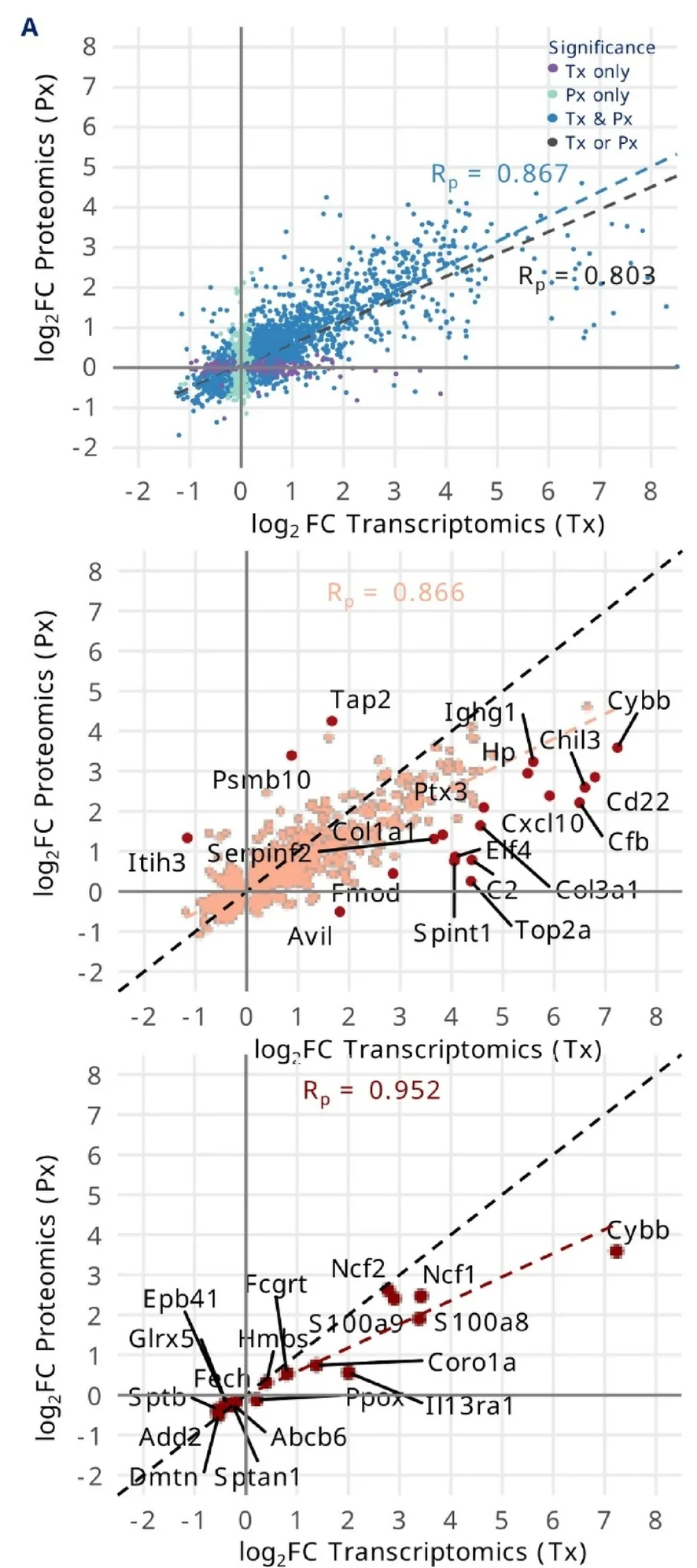
Proteogenomic concordance of differential expression between transcriptomics & proteomics. Scatterplots show log2FCs in transcriptomics (x-axis) vs proteomics (y-axis) for significantly differentially expressed transcripts/proteins, the Pearson correlation coefficient (Rp) & associated *p* value are shown. **A** All genes are coloured by significance: transcriptomics (violet)/proteomics only (turquoise), or both (blue; *p*-adj. < 0.05). Linear regression lines were fitted using significant gene product changes at both levels (blue, Rp = 0.867, *p* < 2.2e-16, CI [0.859, 0.874]) or in at least one level (dark grey, Rp = 0.803, *p* < 2.2e-16, CI [0.795, 0.811]); **B** Corresponding analysis restricted to genes encoding secreted proteins (Rp = 0.866, *p* < 2.2e-16, CI [0.851, 0.88]). Features with the largest discordance between transcriptomic & proteomic log2FCs are annotated with gene symbols. **C** Corresponding analysis restricted to proteins expected to be absent from the healthy murine CNS (Rp = 0.952, *p* = 1.2e-9, CI [0.873, 0.982]); circles annotated with gene symbols

Including genes with non-significant *p*-values in this comparison would lead to inclusion of low confidence non-significant fold changes. Therefore, we performed parallel log_2_FC analyses on two overlapping subsets: (I) gene product changes significant at either one or both molecular levels, and (II) gene product changes significant at both levels. We observed a strong correlation of proteogenomic changes in both cases (Fig. 4A, subset (I): R_p_ = 0.867, *p* < 2.2 × 10⁻1⁶, 95% CI [0.859, 0.874], all datapoints and dark blue regression line; subset (II): R_p_ = 0.803, *p* < 2.2 × 10⁻1⁶, 95% CI [0.795, 0.811], dark grey regression line). The correlation of all gene products identified on both levels including non-significant changes was however comparable (Additional File 1: Fig. S5A). For additional statistical analysis of the correlation, we have implemented a permutation null model using randomly matched log_2_FCs demonstrating significance of observed correlation (Additional File 1: Fig. S5B).

Since GSEA revealed that the pathway *Dengue2 Interactions with Complement and Coagulation Cascades* was upregulated at the protein level only, we hypothesized that secreted proteins may potentially exhibit discordant dysregulation due to compromised BBB integrity. Hence, we performed a correlation analysis specifically for secreted proteins. Interestingly we once again observed a strong correlation of transcript and protein log_2_FCs (R_p_ = 0.866, *p* < 2.2 × 10⁻1⁶, CI [0.851, 0.881], Fig. 4B), with several gene products demonstrating an even higher log_2_FCs at the transcript compared to protein level. On the other hand, we observed higher proteomic upregulation of Tap2, Psmb10, and Itih3 compared to transcriptomic changes. To determine if systemic dysregulation from the periphery biased our CNS-focused findings through protein influx, we assessed the presence of a curated set of 80 genes (Additional File 7: Table S8) which are typically absent in the healthy murine CNS. This set included on the one hand markers for cells which are known to actively infiltrate the SC in the EAE model e.g., T cells, neutrophils, eosinophils and basophils, and on the other hand markers of blood-resident cells which might enter the tissue by passive leakage due to BBB disruption, i.e. erythrocyte and megakaryocyte/platelet. We differentiate between passive leakage which may present as protein-only changes, from cellular infiltration or inflammation-induced upregulation in CNS-resident cells which would be evidenced by the concomitant upregulation of both transcripts and proteins. Out of these 80 gene products, we identified 13 exclusively at the transcript level and only 3 exclusively at the protein level, suggesting limited passive plasma leakage which would be mainly reflected by protein-only changes. However, 18 gene products were identified at both levels, indicating that these gene products may stem from actively infiltrating immune cells or local inflammatory changes bringing both transcripts and proteins into the CNS. To validate this hypothesis, we performed a functional evaluation as well as directly compared their log_2_FCs (Fig. 4C, R_p_ = 0.952, *p* = 1.2 × 10^−9^, CI [0.873, 0.982]). Markers relevant to neutrophil function and immune cell recruitment (e.g., S100a8) showed higher transcript than protein abundance and at the same time higher presence in EAE samples (Additional File 8: Table S9). Similarly, we observed one set of lowly abundant proteins (e.g., Hmbs) with low corresponding transcript expression and comparable presence in naïve and EAE samples. These are relevant for haem biosynthesis, erythrocyte functionality, and the coagulation cascade, suggesting that blood contamination had a limited contribution to the observed datasets, independent of the disease induction.

In summary, the integrated analysis revealed a strong overall correlation between transcriptomic and proteomic fold changes. Secreted proteins exhibited a similarly strong correlation, despite some notable discrepancies in specific genes, which were linked to immune responses, tissue repair, and cellular stress.

## Discussion

We profiled the proteogenomic landscape of the SC and investigated differential expression of transcripts and proteins during acute EAE. While our integrated analysis confirmed well-known signatures of neuroinflammation, we also revealed a strong coherence between transcriptomic and proteomic changes despite model-specific increased BBB permeability.

Overall, differential expression across molecular levels was profoundly skewed towards upregulation, which is in-line with other reports [41] and reflects immune cell infiltration into the CNS and the initiation of neuroinflammatory processes involving local glial activation [7, 42, 43]. We confirmed strong upregulation of gene products related to activated astrocytes, which was previously described after lipopolysaccharide administration in mice as well as during acute EAE [25, 30]. Similarly, we observed a pronounced positive enrichment of pathways linked to microglia and macrophage activation, e.g., interferon-stimulated genes such as Ifitm3 [32, 44]. We also detected pronounced upregulation of Ptprc (CD45), highlighting infiltration of leukocytes into the SC during EAE, a major pathomechanism in the model [45]. GSEA using cell type signature gene sets from MSigDB further validated these findings, revealing significant enrichment for neuronal, microglial, and myeloid signatures in both transcriptomic and proteomic datasets. The CNS infiltration of T cells was evidenced by extensive upregulation of Ahnak [46], although this might also reflect endothelial cell response to BBB disruption [33]. Both observations are in-line with the strong positive enrichment of pathways related to innate and adaptive immunity, including immune signalling, at both molecular levels. Simultaneously, we observed upregulation of immunomodulators such as Serpina3n, which is relevant during astrogliosis [3], Lag3, expressed on immunoregulatory Tr1 cells [23], and Pik3ap1, an inhibitor of TLR signalling [47]. Together, these findings represent a concordant proteogenomic signature of glial activation, immune cell infiltration into the CNS, neuroinflammatory processes, as well as resolving mechanisms following neuropathology, which are expected at the acute time point assessed in this study.

Downregulation indicated metabolic dysfunction during neuroinflammation and was represented by cross-level negative enrichment of pathways linked to mitochondrial assembly and energy metabolism, accompanied by breakdown of the electron transport chain [48, 49] and decreased abundance of gene products like Hsd11b1, a glial- and neuronal-specific protein involved in steroid metabolism [32]. Downregulated signatures highlight key consequences of inflammation in the murine CNS during EAE: we observed pronounced decrease in transcripts and proteins involved in maintenance of the BBB or highly expressed in either the choroid plexus epithelium (*Kirrel2*) [50] or neurons (Ttyh1) [38]. In-line, we observed concordant negative enrichment of pathways linked to synaptic signalling and neuronal functionality across both molecular levels.

Despite these neuroinflammation-characteristic signatures indicating BBB breakdown, which potentially uncouples the CNS-resident transcriptome and proteome [51], we identified a high pathway concordance between mRNA and proteins. Excessive metabolic demand leading to insufficient protein synthesis, BBB disruption, and temporal lag between transcriptomic and proteomic turnover was expected to result in pronounced discrepancies between transcriptomic and proteomics dysregulation. Our findings suggest the presence of counterbalancing mechanisms.

Such mechanisms may have led to transcript-independent upregulation of proteins relevant to inflammatory and cellular stress responses. This pattern is characteristic for inflammation-triggered post-transcriptional and post-translational regulation mechanisms which influence translation efficiency or protein turnover, reflecting adaptations in cellular energy metabolism [48, 52, 53]. Consistent with this, we observed higher proteomic upregulation of Tap2, Psmb10, and Itih3 compared to transcriptomic changes, which may reflect such regulatory events rather than passive trafficking. Furthermore, inflammation induces significant protease activity, such as matrix metalloproteinases and granzymes [54]. These enzymes lead to the generation of protein fragments that may retain functional activity. Moreover, because peptide-based proteomics could capture these fragments, the obtained proteome may represent a mix of full-length and truncated proteins, thereby complicating interpretations. Additionally, ribosomal protein pathways were more strongly enriched than pathways linked to ribosomal transcription, and pathways related to cell cycle and translation were exclusively enriched at the protein level. This pattern may reflect broader translational reprogramming during inflammation such as the potential involvement of the mTORC1 pathway, whose activation leads to increased translational turnover in infiltrating T cells and activated glia. This is supported by the pronounced upregulation of Parp9 in proteomics, which was described to play a role in the mTORC1/PIK3 pathway during neuroinflammation [35]. The same mechanism is likely at play also during EAE, contributing to the unexpectedly high proteogenomic concordance.

In addition, we observed transcript-independent downregulation of proteins involved in cellular integrity and function, as well as negative enrichment of synapse signalling/architecture, cytoskeletal, and structural scaffold pathways. This reflects the extensive breakdown of the CNS architecture including BBB, choroid plexus, as well as neuronal and synaptic network, suggesting that, unlike immune signalling, protein translation to restore the CNS architecture is not prioritized during acute EAE. Similar functional prioritization patterns showing sustained immune signalling alongside reduced homeostatic functions have been described in astrocytes and microglia during neuroinflammation [22, 55]. Of note, while our analysis focuses on the directionality and significance of the enrichment rather than absolute score magnitude, we acknowledge that direct cross-platform comparison of NES values is constrained by differences in dataset depth, feature space, and dynamic range.

The comparison of log_2_FCs revealed a high correlation between regulation on transcript and protein level for the majority of the genes. Most of the discrepancies between log_2_FCs depicted higher values at the transcript level. While our analysis of secreted proteins confirmed a strong correlation, supporting the hypothesis that BBB integrity is relatively maintained in the model, this interpretation relies on canonical database annotations. Such databases often fail to capture proteins with 'moonlighting' functions or dynamic changes under inflammatory stress and therefore further analysis would be required to prove the localization of these proteins in the CNS of EAE mice. Additionally, despite minimizing TMT ratio compression by using MS3 quantification, it may have altered the identified FC on protein level, potentially underestimating the extent of passive leakage. Local, inflammation-driven changes in protein synthesis efficiency or half-life may have influenced our findings as well. Consequently, we cannot definitively exclude the presence of protein trafficking across the BBB, highlighting the need for complementary experimental approaches to validate these observations. However, our analysis of CNS-absent markers revealed low abundance of proteins relevant for haem biosynthesis, erythrocyte functionality, and the coagulation cascade, suggesting only minor blood contamination in all samples. This finding is in opposition with expected plasma protein diffusion across a dysfunctional BBB, which would have led to higher log_2_FCs at the protein level in the absence of transcriptional changes, as reported in other datasets [56].

While our initial analysis of markers typically absent in the healthy CNS mainly identified changes attributed to infiltrating immune cells, it is also known that the inflammatory environment shifts CNS-resident glial cells to a more pro-inflammatory phenotype. Consequently, these markers may be viewed as indicators of the broader EAE-associated tissue state rather than being exclusive to infiltrating populations.

A technical aspect that is typically expected to contribute to discordant dysregulation in proteogenomic analyses is the higher sensitivity of transcriptomics compared to proteomics, as well as differences in sampling and data processing. The uniformed approach applied in our analysis could partially overcome these technical limitations allowing higher molecular correlation. In addition, TMT-based quantification and extensive pre-fractionation lead to more confident identification and quantification of protein across biological replicates.

We identified approximately three times more dysregulated genes at the transcript level compared to the protein level. Many of the genes identified uniquely at the transcript level either showed lower absolute expression compared to those identified at both molecular levels, suggesting challenges with identification of lowly abundant proteins [15, 20], or were highly expressed transcripts belonging to membrane-associated proteins, a class particularly challenging for identification using traditional proteomics approaches (supplemental Fig. S4D) [57].

Another limitation of the current study is the use of only one animal model, one specific clinical timepoint, as well as a limited number of biological replicates. Additional studies with larger sample sizes would be required for definitive exclusion of sex-specific biological effects. Furthermore, the absence of a control group treated with complete Freund's adjuvant and pertussis toxin in the absence of MOG represents a study limitation. We cannot strictly decouple the observed findings from potential non-specific effects of adjuvant components. These restrictions should be considered when interpreting and translating our results.

In conclusion, the integrated analysis employed in our study provides a comprehensive proteogenomic profile of the murine SC during EAE. Our characterization suggests a concordant induction of the immune response in the presence of metabolic stress. In contrast, the relatively minor discordant signatures observed in our study revealed synaptic proteins to be more strongly affected by neurodegenerative mechanisms than corresponding transcripts, deepening the mechanistic understanding of proteogenomic interplay during EAE. Finally, our analysis of log_2_FCs correlation offers a different perspective on the current understanding of extensive protein trafficking across the BBB during neuroinflammation and offers a resource to investigate location and origin of drug targets in neuroinflammation.

## Supplementary Information

Below is the link to the electronic supplementary material.

Supplementary Material 1

Supplementary Material 2

Supplementary Material 3

Supplementary Material 4

Supplementary Material 5

Supplementary Material 6

Supplementary Material 7

Supplementary Material 8

Supplementary Material 9

Supplementary Material 10

## Data Availability

The datasets supporting the conclusions of this article are included within the article and its additional files. The transcriptomic and proteomic datasets generated during this study are available in the following repositories: RNA-seq data are deposited in the NCBI Gene Expression Omnibus (GEO) under accession number GSE330115 (https://www.ncbi.nlm.nih.gov/geo/query/acc.cgi?acc=GSE330115). The mass spectrometry proteomics data have been deposited to the ProteomeXchange Consortium via the PRIDE partner repository with the dataset identifier PXD078146 and https://doi.org/10.6019/PXD07814.

## Abbreviation

ADHD: Attention deficit hyperactivity disorder
ASD: Autism spectrum disorder
BBB: Blood–brain barrier
BCAP: B-cell adaptor for phosphoinositide 3-kinase
BCA: Bicinchoninic acid
CD45: Protein tyrosine phosphatase receptor type C (Ptprc)
CID: Collision-induced dissociation
CPM: Counts per million
CNS: Central nervous system
DEG: Differentially expressed gene
DEP: Differentially expressed protein
dpi: Days post-immunization
EAE: Experimental autoimmune encephalomyelitis
ECM: Extracellular matrix
FA: Formic acid
FDR: False discovery rate
FSGS: Focal segmental glomerulosclerosis
GABA: Gamma-aminobutyric acid
GSEA: Gene set enrichment analysis
HCD: Higher-energy collisional dissociation
HPLC: High-performance liquid chromatography
IFN: Interferon
IL: Interleukin
iST: In-StageTip
LC–MS/MS: Liquid chromatography–tandem mass spectrometry
log_2_FC: Log_2_ fold change
MAPK: Mitogen-activated protein kinase
MOG: Myelin oligodendrocyte glycoprotein
mRNA: Messenger RNA
MS: Multiple sclerosis
MS1: First stage of mass spectrometry
MS2: Second stage of mass spectrometry
MS3: Third stage of mass spectrometry
mTORC1: Mechanistic target of rapamycin complex 1
NES: Normalized enrichment score
NF-κB: Nuclear factor kappa B
NGS: Next-generation sequencing
OxPhos: Oxidative phosphorylation
PBS: Phosphate-buffered saline
PIK3: Phosphoinositide 3-kinase
PSM: Peptide spectrum match
RNA-seq: RNA sequencing
SC: Spinal cord
S/N: Signal-to-noise ratio
SPS: Synchronous precursor selection
TCA: Tricarboxylic acid
TLR: Toll-like receptor
TMT: Tandem mass tag
Tr1: Type 1 regulatory T cell

## Declarations

### Use of artificial intelligence assistance

The authors acknowledge the use of GitHub Copilot to assist in writing/reviewing custom R and Python code. All code was validated by humans, the authors take full responsibility for the analytical output.
