# Supplementary material for: Integrated proteogenomic profiling reveals coordinated differential expression signatures during neuroinflammation
Source: Acta Neuropathol Commun. 2026 Jul 10;14:204. doi: 10.1186/s40478-026-02365-0 (PMC13640074; doi:10.1186/s40478-026-02365-0)
Supplement: Supplementary file 1 [file 40478_2026_2365_MOESM9_ESM.pdf]

Detailed analysis description of harmonized data analysis for transcriptomics and proteomics

Variation partitioning analysis

To quantify the contribution of biological covariates to overall dataset variability, variation partitioning analysis was performed using linear models. The relative contributions of disease state (EAE vs. naïve), sex, and the interaction of disease state–sex were calculated separately for transcriptomic and proteomic datasets. Results were expressed as the proportion of variance explained by each covariate and visualized accordingly (Figure S2).

Calculation of differential expression

Differentially expressed genes (DEG) and differentially expressed proteins (DEP) were computed in R using edgeR and limma. For DEG calculation, we filtered weakly expressed genes to retain genes of a count per million (CPM) thresholds of more than one in at least three samples. Counts were transformed into log2-counts-per-million with precision weights using voom (limma). For DEP calculation, protein intensities were normalized using the Trimmed Mean of M-values method and log2-transformed. Transcriptomic and proteomic analysis followed an identical downstream analysis pipeline: Linear models were fitted with lmFit (limma) and empirical Bayes moderation was performed. Gene-wise statistics and multiple-testing adjusted P values (p-adj) applying Benjamini–Hochberg were obtained using topTable (limma) across all genes. For the contrast EAE vs. naïve (baseline), log2 fold changes ($\log_2$FCs), raw and p-adj (reported as −log10 values for visualization), t statistics, and decision calls from decideTests (limma, applying method="separate", BH adjustment, false discovery rate (FDR) threshold 0.01, and $|\log_2FC| \geq 1$) were summarized in DEG tables.

Gene Set Enrichment Analysis (GSEA)

GSEA was performed using the fgsea package with t statistic-based ranked gene-level statistics. Results obtained using multiple gene set collections (Reactome, Gene Ontology Biological Processes, Gene Ontology Molecular Functions Hallmarks, ImmuneSigDB, WikiPathways, Supplementary Tables S4-13) validated reproducibility of our findings. We display GSEA results using WikiPathways (version per 11.09.25) in the main text to spotlight manually curated,

biologically distinct signalling cascades. Gene sets were curated from Molecular Signatures Database (MSigDB). Fgsea (PermSimple=10,000, minSize=15, maxSize=500) was run and enrichment results were summarized as normalized enrichment scores (NES), P values, and padj (Benjamini–Hochberg). We considered pathway enrichment significant at padj$\leq$0.05. To ensure that similar pathway enrichment was not masked by semantical deviations, GSEA results were condensed by pairwise Jaccard similarity calculations of core genes driving enrichment (leading-edge genes) to cluster pathways via hierarchical clustering with a cutoff of 75% similarity. From each cluster, the most significant pathway was retained as the representative.

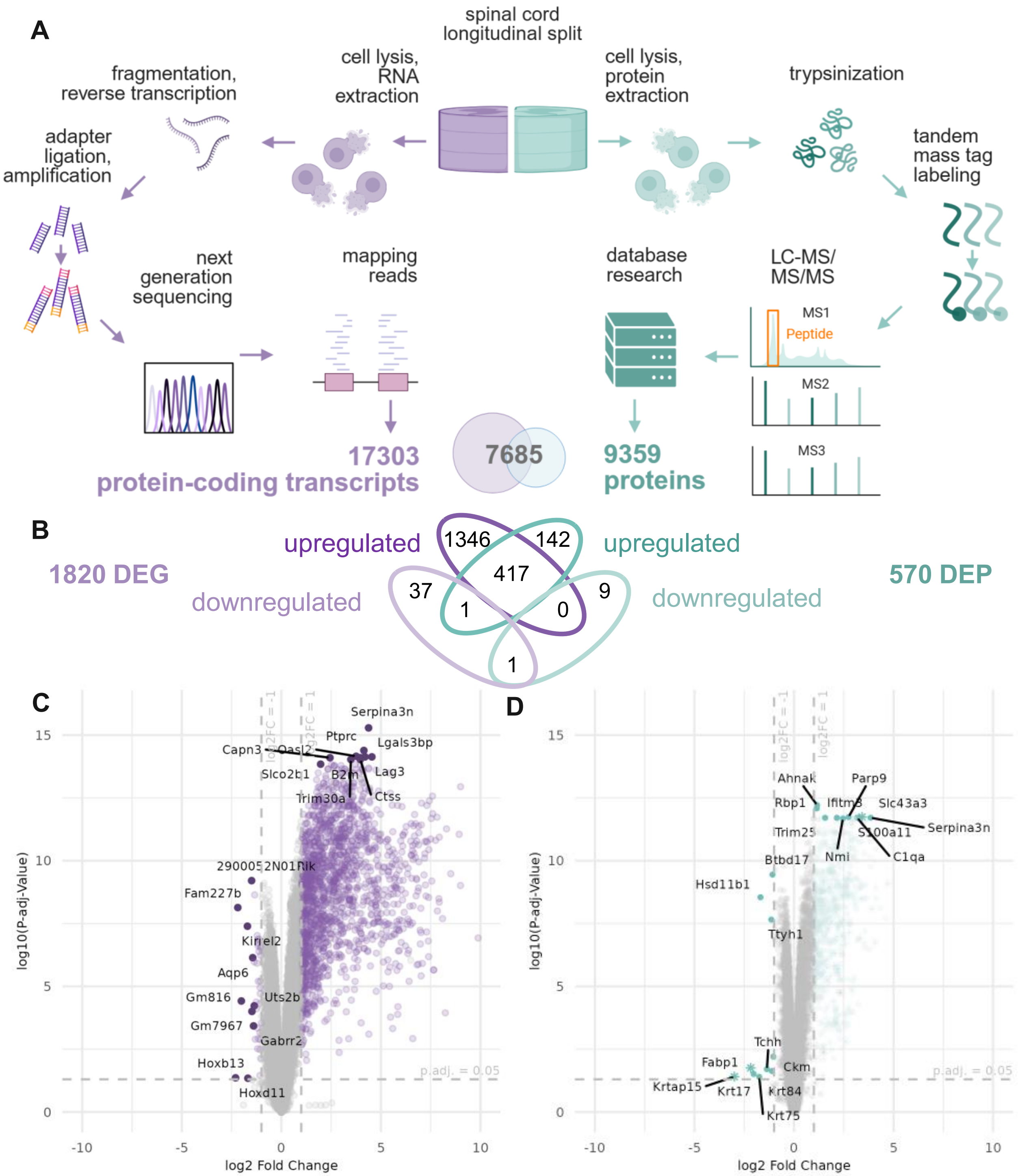

Fig. 1: Proteogenomic profiling of EAE spinal cords reveals predominant upregulation. (**A**) Schematic workflow of combined transcriptomic and proteomic analysis. Spinal cord tissue from EAE & naive control mice was processed in parallel for RNA-sequencing (transcriptomics, violet) and LC-MS/MS-based profiling (proteomics, turquoise). (**B**) Venn diagram illustrating the count of unique and shared significantly (p-adj.<0.05, $log_2FC>1$) differentially expressed transcripts/proteins (DEG/DEP). (**C-D**) Volcano plots displaying the differentially expressed transcripts **(C)** or proteins **(D)**. X-axes represent the log_2FC (EAE vs. naive), y-axes represent the significance level (log_{10} of p-adj.). Coloured circles denote significantly (p-adj.<0.05, $log_2FC>1$) dysregulated genes, the ten candidates with highest absolute t-statistics are labelled. Proteins with significant changes but identified by only one PSM are displayed as asterisks.

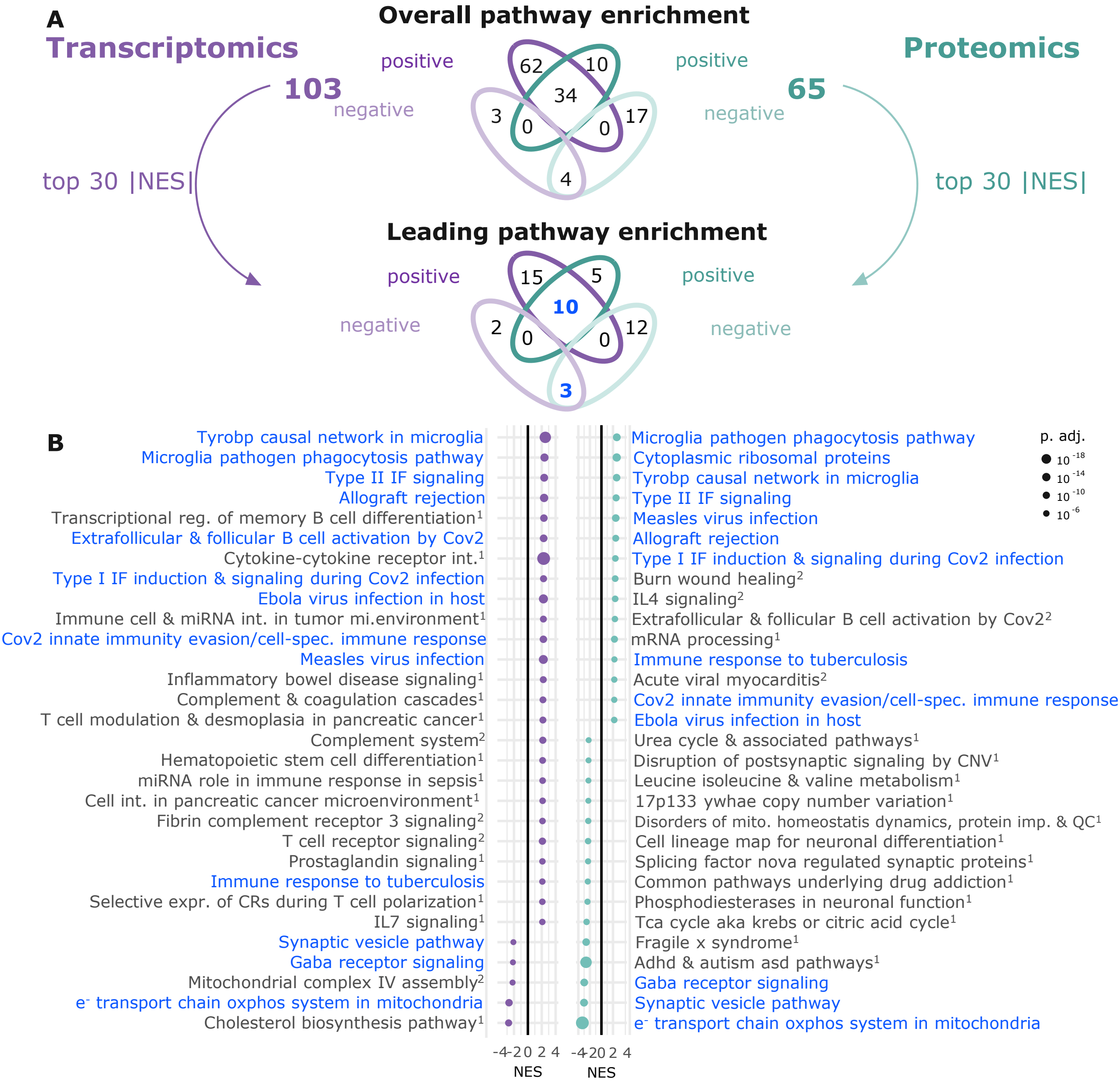

Fig. 2: GSEA reveals concordant proteogenomic enrichment patterns. (A) Venn diagrams illustrating the count of unique & shared significantly enriched pathways (p-adj.<0.05) in transcriptomics (violet) and proteomics (turquoise). (**B**) Top 30 enriched pathways in transcriptomics (left) or proteomics (right). X-axes represent the normalised enrichment score (NES), circle size indicates significance level. Pathways shared between the top 30 hits of both datasets are coloured to visualize convergence. Superscript numbers indicate if non-shared pathways were not enriched ([1]) at the other molecular level or enriched into the same direction ([2]). Cov2: sarscov2, CR: chemokine receptor, expr.: expression, imp.: import, int.: interaction, mi.environment: microenvironment, mito.: mitochondrial, QC: quality control, reg.: regulation.

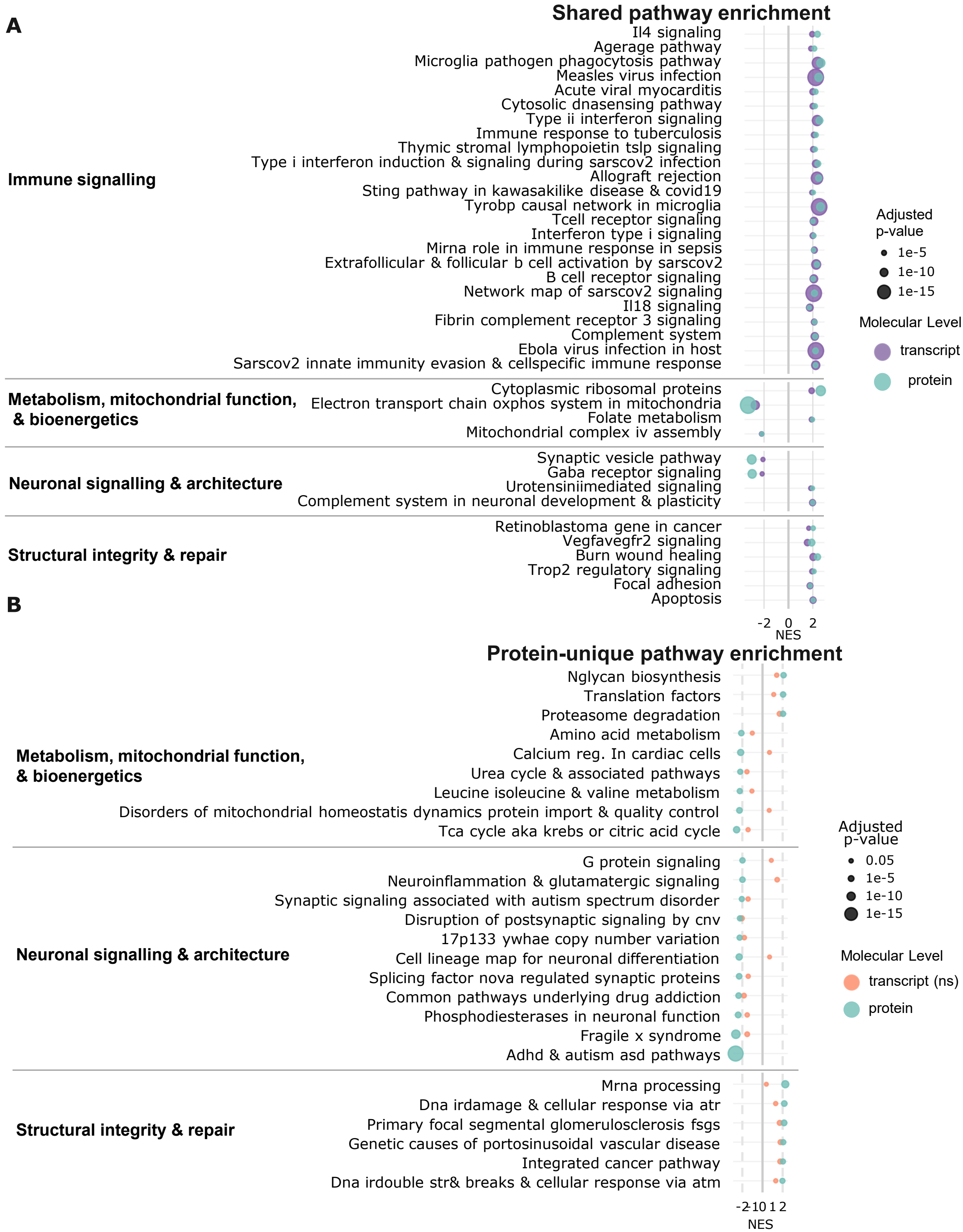

Fig. 3: Comparative GSEA underlines concordant orchestration of the immune response alongside discordant synaptic and mitochondrial maintenance. (**A**) Pathways depicting strongest differences between transcriptomic (violet) & proteomic (turquoise) normalised enrichment scores (NES), sorted highest to smallest. Circle colour denotes the data type (transcript vs protein), and circle size denotes statistical significance. (**B**) Pathways enriched uniquely at the protein level, sorted according to highest NES. The horizontal line separates pathways ranked among top 30 from the remaining ones.

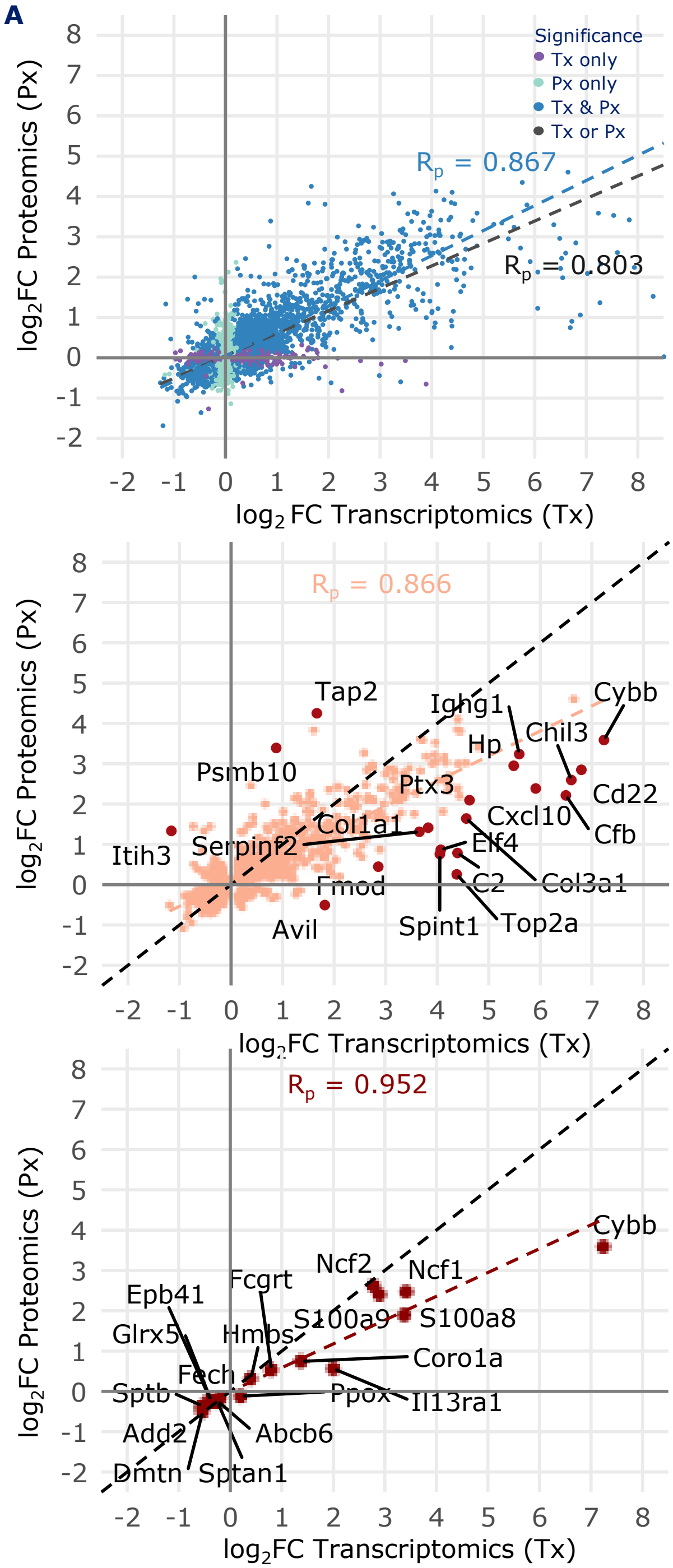

Fig. 4: Proteogenomic concordance of differential expression between transcriptomics and proteomics. Scatterplots show log_2FCs in transcriptomics (x-axis) vs proteomics (y-axis) for significantly differentially expressed transcripts/proteins, the Pearson correlation coefficient (R_p) & associated p value are shown. (**A**) All genes are coloured by significance: transcriptomics (violet) / proteomics only (turquoise), or both (blue; p-adj.<0.05). Linear regression lines were fitted using significant gene product changes at both levels (blue, R_p=0.867, p<2.2e-16, CI [0.859, 0.874]) or in at least one level (dark grey, R_p=0.803, p<2.2e-16, CI [0.795, 0.811]); (**B**) Corresponding analysis restricted to genes encoding secreted proteins (R_p=0.866, p<2.2e-16, CI: [0.851, 0.88]). Features with the largest discordance between transcriptomic and proteomic log_2FCs are annotated with gene symbols. (**C**) Corresponding analysis restricted to proteins expected to be absent from the healthy murine CNS (R_p=0.952, p=1.2e-9, CI: [0.873, 0.982]); circles annotated with gene symbols.

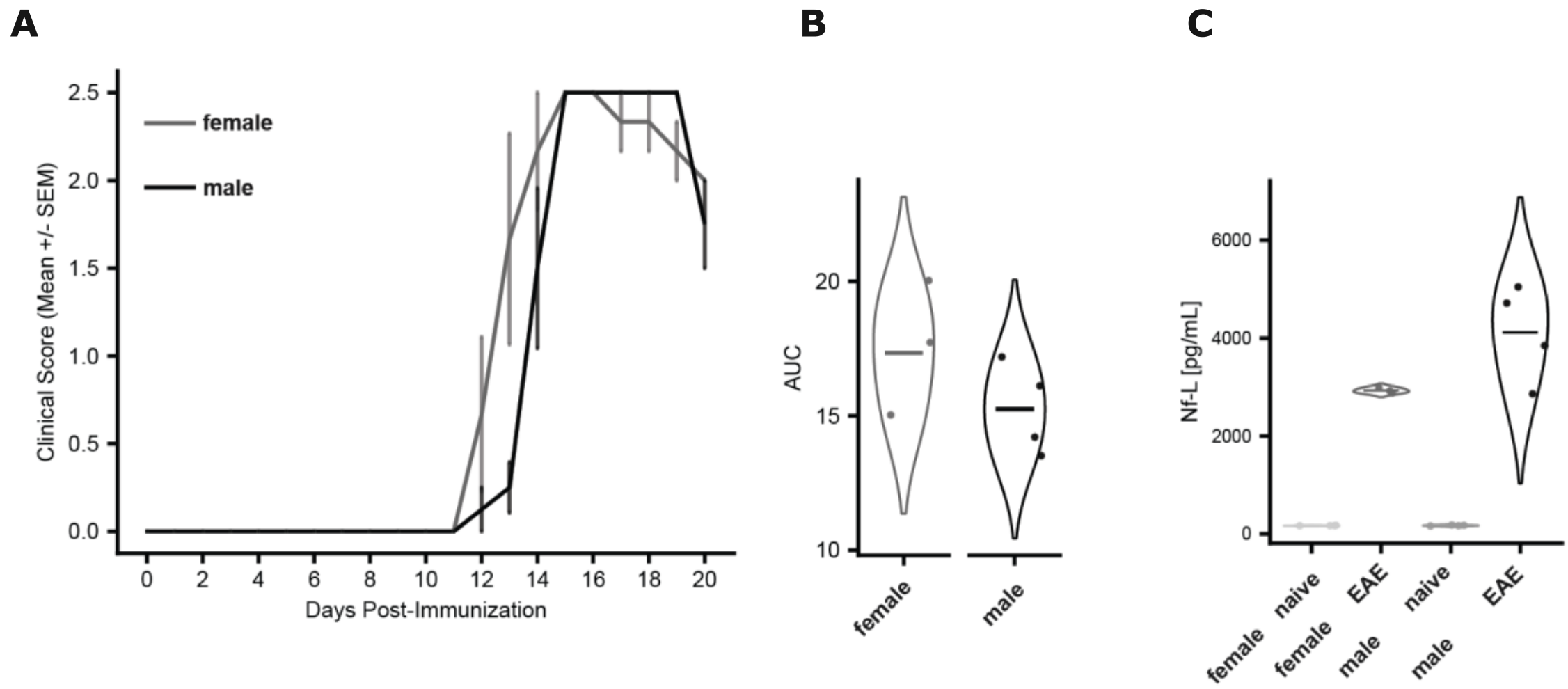

Fig. S1: Clinical characterization of the EAE cohort. (**A**) Daily clinical scores EAE mice (n=7), monitored until the study endpoint. Data are presented as mean ± standard error of the mean (SEM). (**B**) Cumulative disease severity quantified as Area Under the Curve (AUC) from day 0–20 post-immunization. (**C**) Plasma Neurofilament Light Chain (NfL) concentration measured in naive (n=8) and EAE (n=7) mice at the time of sacrifice, serving as a marker of neuroaxonal injury. For (B) & (C), violin plots illustrate the data distribution, with central bars indicating the median & interquartile ranges (IQR).

A

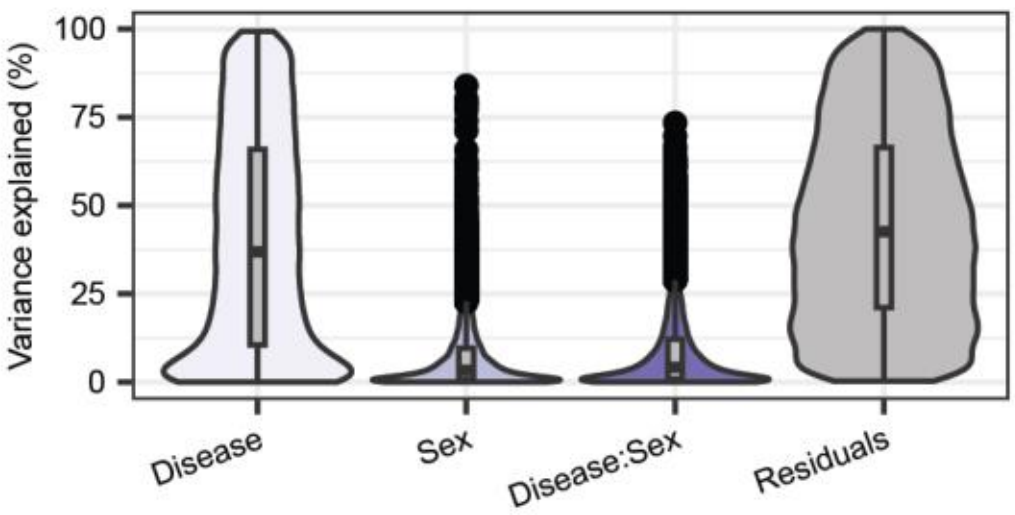

B

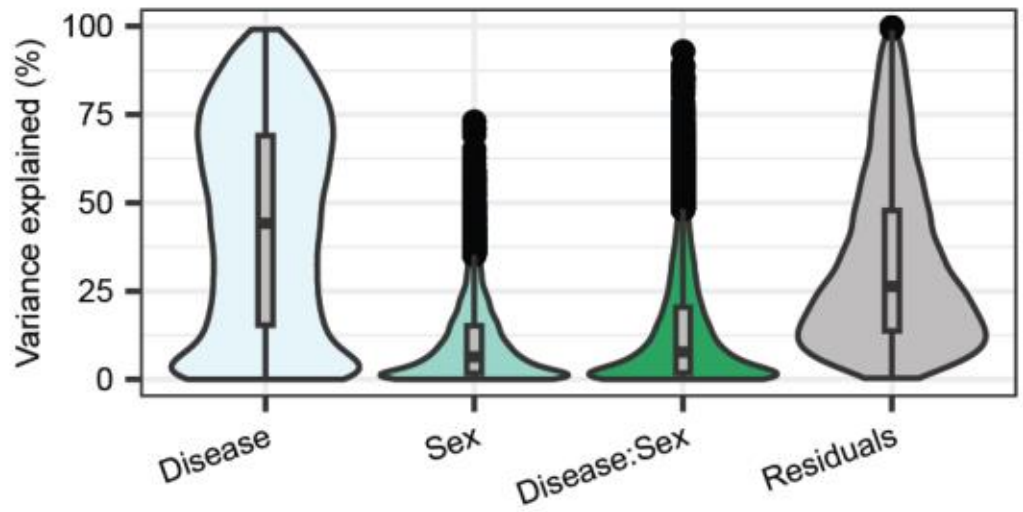

C Table 1: Top Genes Contributing to Gene Variation in Transcriptomics

Disease	Sex	DiseaseSex	Residuals
Ptprc	Eif2s3y	Gnai3	Luzp2
Xcr1	Gm29650	Cdc45	Gas2l1
Zbp1	Ddx3y	H19	Pgam1-ps1
Gm8979	Xist	Scml2	D8Ertd738e
Pik3ap1	Kdm5d	Narf	Duxbl3
Gm8989	Uty	Cav2	Msl3
Serpina3n	Eif2s3x	Klf6	Dhx9
Slco2b1	Kdm6a	Scmh1	Amot
Lag3	5530601H04Rik	Cox5a	Fgfr1
Tlr12	1700030C10Rik	Tbx2	Rps6kb2
Hk2	Eva1a	Ngfr	Gpd1
Slfn8	Amacr	Wnt3	Lsm4
H2-Q7	Trh	Wnt9a	Atp6v0b
Trim30a	Gapdh	Fer	Zfp160
Phf11d	Tfb1m	Xpo6	Duxbl2
Selplg	Gm9917	Tfe3	Dpm2
Xaf1	Stil	Axin2	2900093K20Rik
Capn3	Slc2a5	Brat1	Nmrk1
Vim	Angptl6	Gna12	Zswim6
Oas1g	Mkrn3	Slc22a18	Chmp2a

D Table 2: Top Genes Contributing to Gene Variation in Proteomics

Disease	Sex	DiseaseSex	Residuals
Ahnak	Ddx3y	Gnai3	Phgdh
Aldh1l2	Naa25	Apoh	BC005624
Rbp1	Flot1	Cav2	Mapk11
Ifitm3	Trappc10	Cox5a	Mpp5
C4b	Vps37a	Ngfr	Rtca
Ifit1	Itfg1	Fer	Mep1a
Slc43a3	Tvp23a	Xpo6	Eloc
Osmr	Rab4a	Tfe3	Smyd3
H2-D1	Pcbp4	Brat1	Htt
Ifi35	Ankhd1	Gna12	Scamp4
Gbp9	Coasy	Dlat	Ctu2
Cd44	Nsd2	Sdhd	Cpeb4
C1qa	Flii	Gpr107	Asb3
Tap2	Zfyve9	Btbd17	Ring1
Casp12	Mrpl41	Th	Abca8b
Gimap4	Tfe3	Drp2	Pradc1
Fcgr2b	Usp11	Gmpr	Abcf2
Cd180	Fam151b	Glra1	Dhx57
Rtp4	Dennd1a	Mid2	Clns1a
Pml	Cox6a1	Trim25	Jade2

Fig. S2: Disease state, but not sex, influences dataset variability. (A-B) Variation partitioning analysis of the relative contributions of EAE induction (disease), sex, and the int. of EAE induction and sex to the variability of the transcriptomics (**A**) and proteomics (**B**) datasets. Top 20 genes contributing to the highest extent to factor variability in transcriptomics (**C**) and proteomics (**D**).

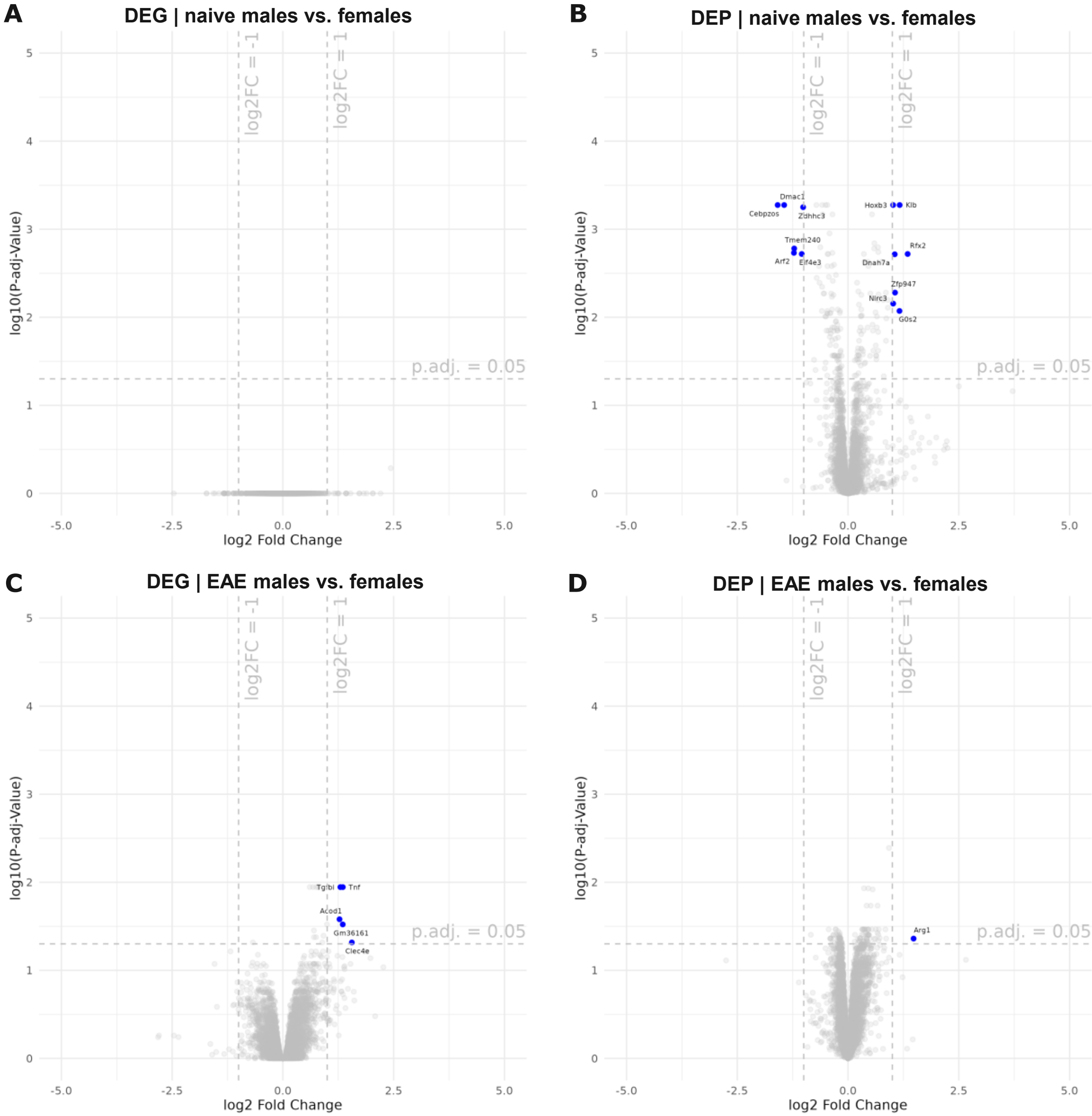

Fig. S3. **(A,B)** Volcano plots displaying differentially expressed genes (DEG) **(A)** & differentially expressed proteins (DEP) in the spinal cord **(B)** comparing naive males against naive females (each n=4). **(C,D)** Volcano plots displaying DEG **(C)** and DEP **(D)** comparing EAE-induced males (n=4) against females (n=3). In all panels, significantly regulated gene products (p.adj < 0.03, log2FC ≥ 1.0) are highlighted in blue and annotated. Females were used as the baseline, positive log2FCs indicate upregulation in males, accordingly. Significance was calculated with limma eBayes and Benjamini-Hochberg FDR correction.

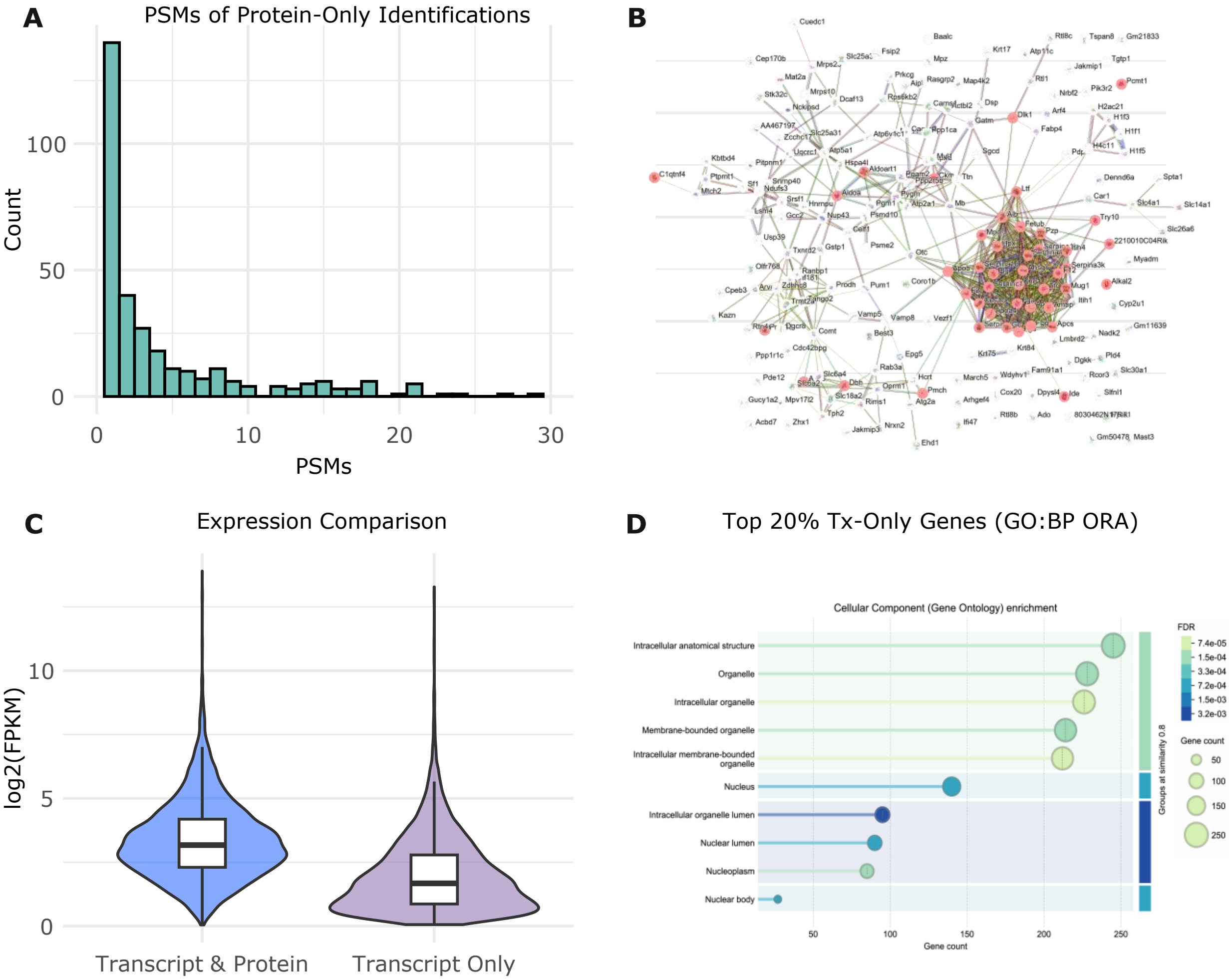

Fig. S4. (**A**) Distribution of PSMs for genes identified only on protein level. (**B**) int. of genes identified only on protein level with more than 1 PSM, the genes from extracellular region (GO:0005576) are highlighted in red. (**C**) Expression values $\log_2$(FPKM) of genes identified on only transcript level versus genes identified on both transcript & protein levels. (**D**) Gene set enrichment on top 20% of the most expressed genes exclusively identified on transcript level, sorted by gene count.

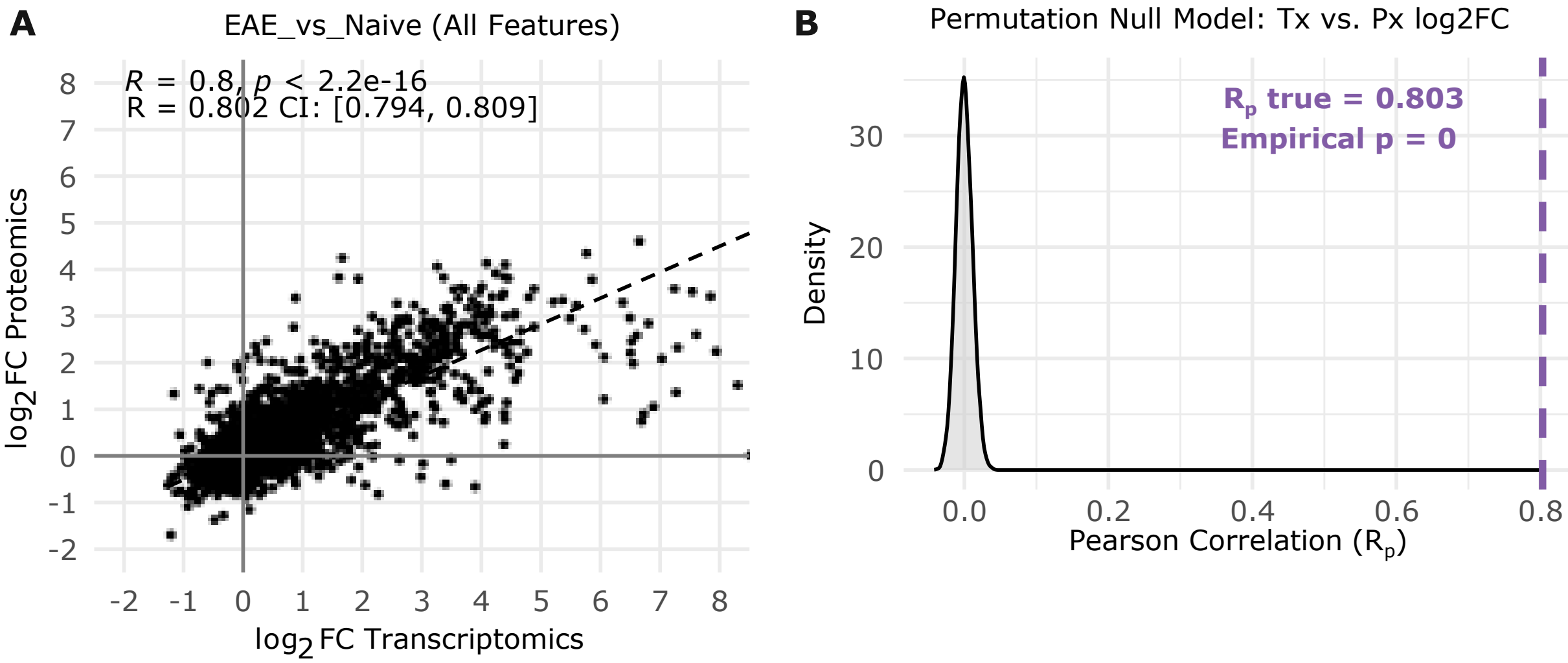

Fig. S5. Mathematical validation of correlation analysis (**A**) Scatterplot showing log_2FCs in transcriptomics (x-axis) vs proteomics (y-axis) for all identified on both gene levels transcripts/proteins. The linear regression line, Pearson correlation coefficient (R_p) and associated p value are shown. (B) Permutation Null Model of Proteogenomic correlation. The grey curve represents the distribution generated via 10,000 random matches of Tx and Px log_2FC values. The dashed line denotes the actual biological correlation (R_p = 0.803) observed in the matched datasets as shown in main Figure 4A (black regression line). The empirical p-value indicates the probability of observing this correlation by chance.

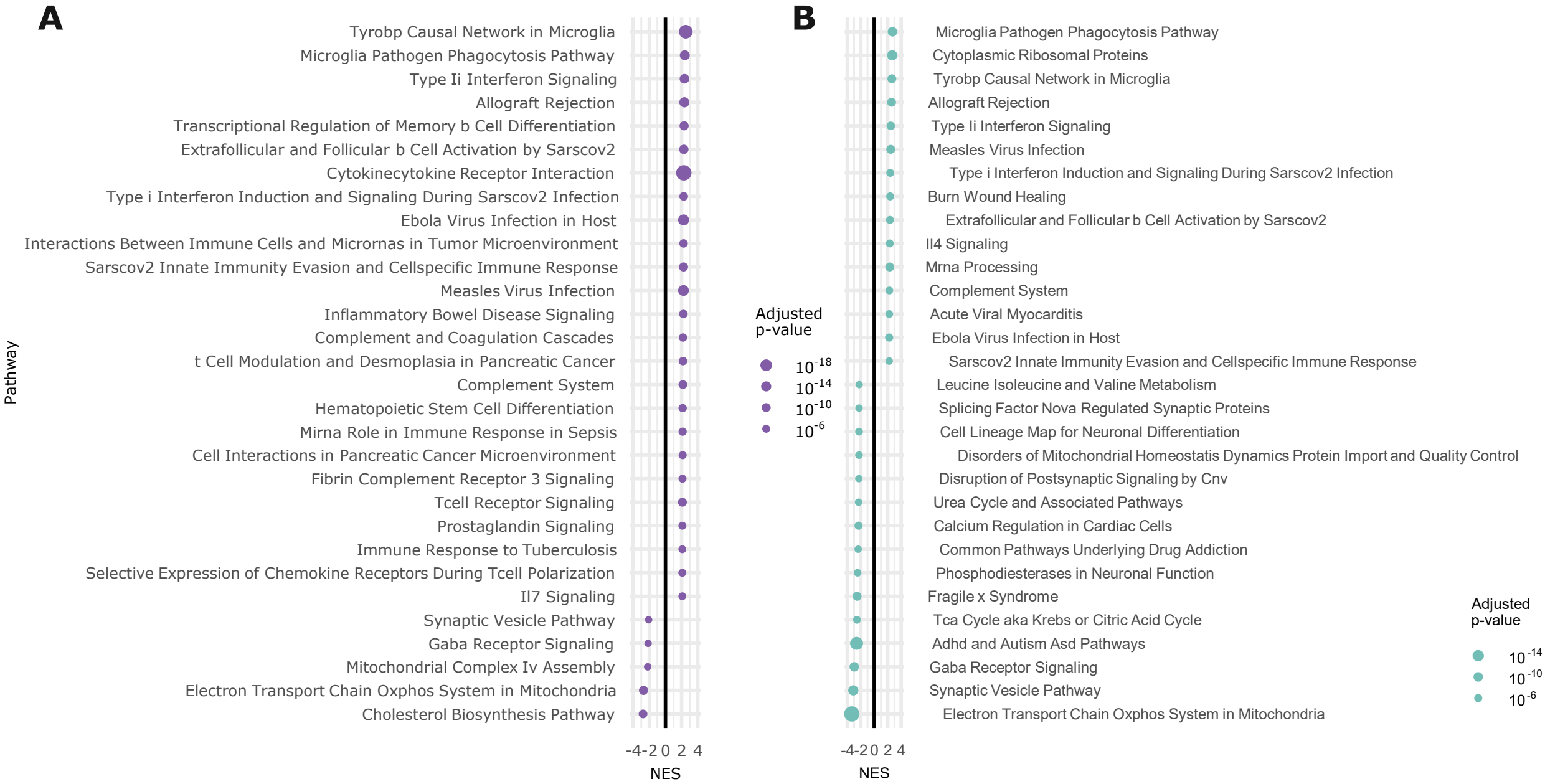

Fig. S6. GSEA results shown in main Fig. 2 after excluding 1 PSM findings in the proteomics dataset. Results of the sensitivity analysis excluding proteins identified by only 1 PSM. All other analytical parameters and representations remain identical to Fig. 1 in the main text.

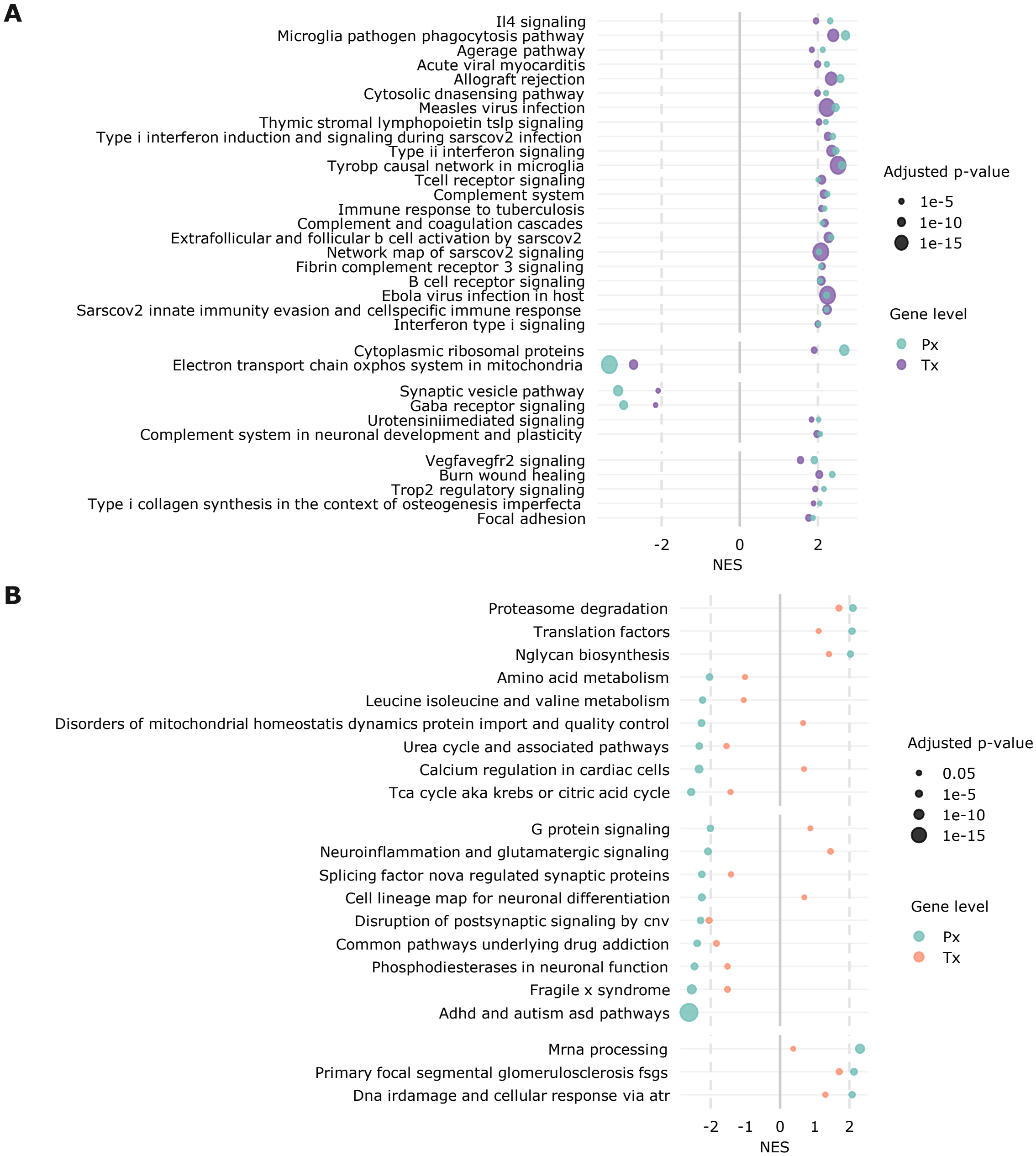

Fig. S7. GSEA results shown in main Fig. 3 after excluding 1 PSM findings in the proteomics dataset. Results of the sensitivity analysis excluding proteins identified by only 1 PSM. All other analytical parameters and representations remain identical to Fig. 3 in the main text.

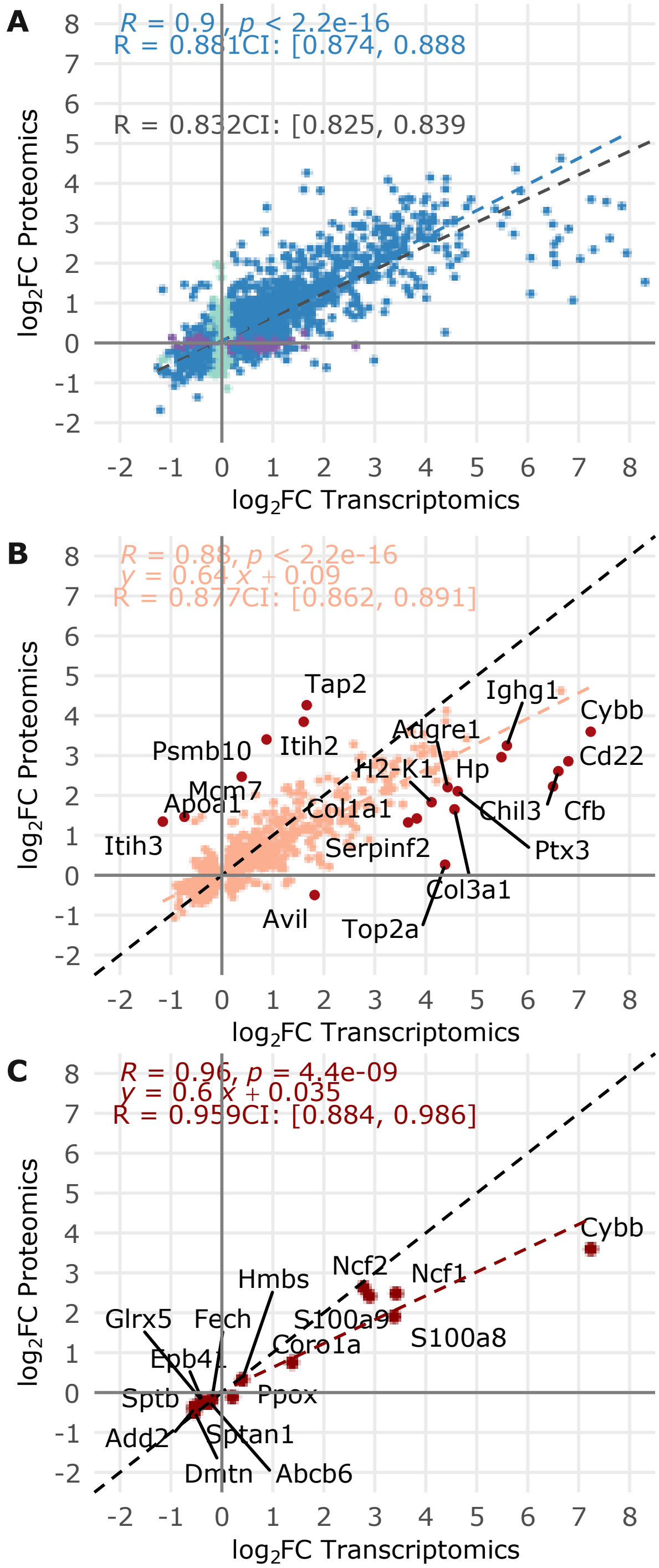

Fig. S8. Correlation results shown in main Fig. 4 after excluding 1 PSM findings in the proteomics dataset. Results of the sensitivity analysis excluding proteins identified by only 1 PSM. All other analytical parameters and representations remain identical to Fig. 4 in the main text.
