# Supplementary material for: Integrated proteogenomic profiling reveals coordinated differential expression signatures during neuroinflammation
Source: Acta Neuropathol Commun. 2026 Jul 10;14:204. doi: 10.1186/s40478-026-02365-0 (PMC13640074; doi:10.1186/s40478-026-02365-0)
Supplement: Supplementary file 2 [file 40478_2026_2365_MOESM1_ESM.docx]

Tables

| **Table S8:** Annotated panel of 80 blood-specific, CNS-silent marker genes grouped by hematopoietic lineage and function | |
| --- | --- |
| Category | Genes |
| Erythrocyte-specific | Membrane proteins:  Slc4a1, Epb42, Gypa, Gypb, Kel, Rhag, Add2, Sptan1, Sptb, Epb41, Epb49, Gypc, Darc, Icam4, Rhce, Kell  Heme biosynthesis:  Alas2, Hemgn, Slc25a37, Urod, Uros, Ppox, Hmbs, Alad, Glrx5, Abcb6, Cpox, Abcb10, Fech  Cytoskeletal:  Dmtn, Tmod1 |
| Neutrophil-specific | Ly6g, Ngp, Elane, Prtn3, Mpo, Camp, Fcgr3b, Fcgr2a, S100a8, S100a9, Csf3r, Il1r2, Il1rn, Il8rb, Il13ra1, Fcgrt, C3ar1, Fpr1, Emr2, Ncf1, Ncf2, Cybb, Cyba |
| Eosinophil-specific | Ear2, Siglecf, Il5ra, Prg2, Prg3, Epx, Ccr3, Ear1, Mkx |
| Basophil-specific | Ms4a2, Fcer1a, Cpa3, Mcpt8, Tpsab1, Hdc |
| Megakaryocyte/Platelet-specific | Pf4, Ppbp, Itga2b, Gp9, Gp5, Mpl, Gp1ba, Gp1bb |
| Lymphoid/Myeloid progenitor | Coro1a, Ms4a3, Trim58 |
